# Longitudinal metabolite and protein trajectories prior to diabetes mellitus diagnosis in Danish blood donors: a nested case–control study

**DOI:** 10.1007/s00125-024-06231-3

**Published:** 2024-07-30

**Authors:** Agnete T. Lundgaard, David Westergaard, Timo Röder, Kristoffer S. Burgdorf, Margit H. Larsen, Michael Schwinn, Lise W. Thørner, Erik Sørensen, DBDS Genomic Consortium, Kaspar R. Nielsen, Henrik Hjalgrim, Christian Erikstrup, Bertram D. Kjerulff, Lotte Hindhede, Thomas F. Hansen, Mette Nyegaard, Ewan Birney, Hreinn Stefansson, Kári Stefánsson, Ole B. V. Pedersen, Sisse R. Ostrowski, Peter Rossing, Henrik Ullum, Laust H. Mortensen, Dorte Vistisen, Karina Banasik, Søren Brunak

**Affiliations:** 1 Novo Nordisk Foundation Center for Protein Research, Faculty of Health and Medical Sciences, University of Copenhagen, Copenhagen, Denmark; 2 Methods and Analysis, Statistics Denmark, Copenhagen, Denmark; 3 The Recurrent Pregnancy Loss Unit, Copenhagen University Hospitals Rigshospitalet and Hvidovre, Copenhagen, Denmark; 4 Department of Clinical Immunology, Copenhagen University Hospital, Rigshospitalet, Copenhagen, Denmark; 5 Department of Clinical Immunology, Aalborg University Hospital, Aalborg, Denmark; 6 Danish Cancer Society Research Center, Copenhagen, Denmark; 7 Department of Epidemiology Research, Statens Serum Institut, Copenhagen, Denmark; 8 Department of Haematology, Copenhagen University Hospital, Rigshospitalet, Copenhagen, Denmark; 9 Department of Clinical Medicine, Copenhagen University, Copenhagen, Denmark; 10 Department of Clinical Immunology, Aarhus University Hospital, Aarhus, Denmark; 11 Department of Clinical Medicine, Health, Aarhus University, Aarhus, Denmark; 12 Department of Neurology, Copenhagen University Hospital - Rigshospitalet, Glostrup, Denmark; 13 Department of Health Science and Technology, Faculty of Medicine, Aalborg University, Aalborg, Denmark; 14 European Molecular Biology Laboratory, European Bioinformatics Institute, Cambridge, UK; 15 deCODE Genetics, Reykjavik, Iceland; 16 Department of Clinical Medicine, Faculty of Health and Medical Sciences, University of Copenhagen, Copenhagen, Denmark; 17 Department of Clinical Immunology, Zealand University Hospital, Køge, Denmark; 18 Steno Diabetes Center Copenhagen, Herlev, Denmark; 19 Statens Serum Institut, Copenhagen, Denmark; 20 Department of Public Health, University of Copenhagen, Copenhagen, Denmark; 21 Novo Nordisk A/S, Bagsværd, Denmark

**Keywords:** Molecular biomarkers, Multi-omics, Polygenic risk scores, Temporality, Time-to-event prediction, Type 1 diabetes mellitus, Type 2 diabetes mellitus

## Abstract

**Aims/hypothesis:**

Metabolic risk factors and plasma biomarkers for diabetes have previously been shown to change prior to a clinical diabetes diagnosis. However, these markers only cover a small subset of molecular biomarkers linked to the disease. In this study, we aimed to profile a more comprehensive set of molecular biomarkers and explore their temporal association with incident diabetes.

**Methods:**

We performed a targeted analysis of 54 proteins and 171 metabolites and lipoprotein particles measured in three sequential samples spanning up to 11 years of follow-up in 324 individuals with incident diabetes and 359 individuals without diabetes in the Danish Blood Donor Study (DBDS) matched for sex and birth year distribution. We used linear mixed-effects models to identify temporal changes before a diabetes diagnosis, either for any incident diabetes diagnosis or for type 1 and type 2 diabetes mellitus diagnoses specifically. We further performed linear and non-linear feature selection, adding 28 polygenic risk scores to the biomarker pool. We tested the time-to-event prediction gain of the biomarkers with the highest variable importance, compared with selected clinical covariates and plasma glucose.

**Results:**

We identified two proteins and 16 metabolites and lipoprotein particles whose levels changed temporally before diabetes diagnosis and for which the estimated marginal means were significant after FDR adjustment. Sixteen of these have not previously been described. Additionally, 75 biomarkers were consistently higher or lower in the years before a diabetes diagnosis. We identified a single temporal biomarker for type 1 diabetes, IL-17A/F, a cytokine that is associated with multiple other autoimmune diseases. Inclusion of 12 biomarkers improved the 10-year prediction of a diabetes diagnosis (i.e. the area under the receiver operating curve increased from 0.79 to 0.84), compared with clinical information and plasma glucose alone.

**Conclusions/interpretation:**

Systemic molecular changes manifest in plasma several years before a diabetes diagnosis. A particular subset of biomarkers shows distinct, time-dependent patterns, offering potential as predictive markers for diabetes onset. Notably, these biomarkers show shared and distinct patterns between type 1 diabetes and type 2 diabetes. After independent replication, our findings may be used to develop new clinical prediction models.

**Graphical Abstract:**

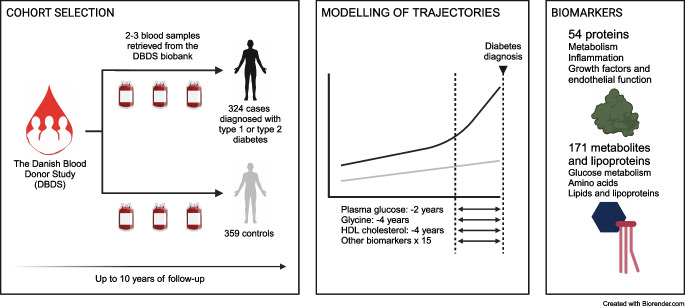

**Supplementary Information:**

The online version contains peer-reviewed but unedited supplementary material available at 10.1007/s00125-024-06231-3.


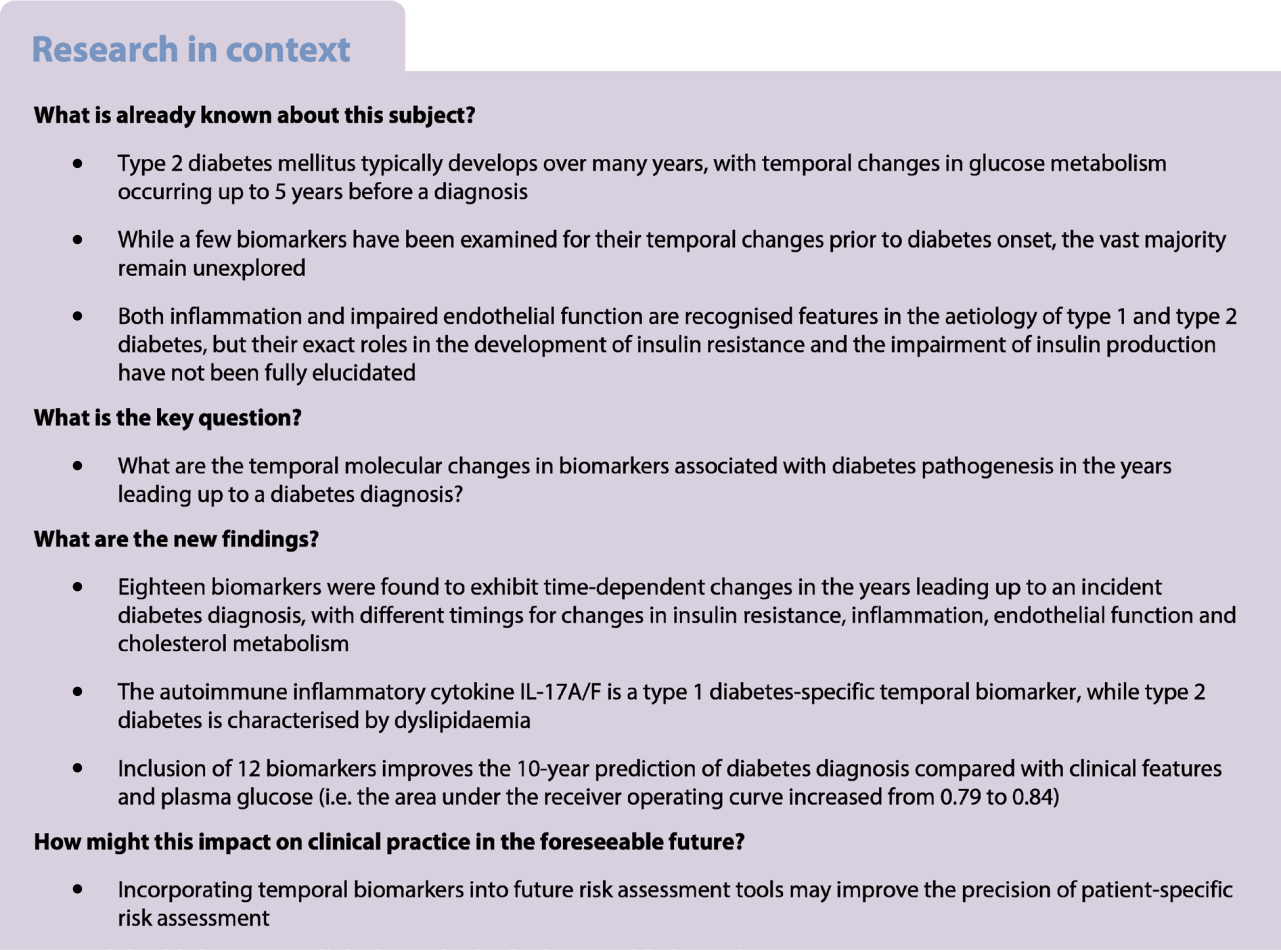


## Introduction

The ongoing increase in the prevalence of type 2 diabetes mellitus poses a need for better diagnostics and treatment of the disease [1]. Timely detection of the onset of type 2 diabetes is crucial to reduce the harmful effects of long-term hyperglycaemia and hyperinsulinaemia on tissues such as blood vessels and nerves [2, 3]. The inclusion of molecular biomarkers in predictive models for type 2 diabetes diagnosis has been shown to improve accuracy compared with models based exclusively on risk factors such as family history, lifestyle behaviour and anthropometric measures [4–6]. Moreover, using combinations of biomarkers in panels may be more effective than using single biomarkers [5]. The discovery of novel biomarkers may also provide new insights into disease mechanisms, yield more accurate risk estimates for type 2 diabetes to initiate screening and prevention measures, and be used in identification of targets for new treatments [4, 7, 8].

Previous studies have described the temporal changes in biomarkers such as BMI, fasting glucose, LDL, HDL, triacylglycerol and C-reactive protein (CRP) preceding a type 2 diabetes diagnosis [9–14]. These studies show a clear trend for an increasing burden of risk factors towards the time of diagnosis. However, these temporal biomarkers only cover a small subset of the biomarkers that have been reported to be associated with diabetes mellitus [7]. Moreover, inflammation, a central driver of diabetes [15, 16], is poorly covered by current biomarkers, except for CRP [9, 13] and IL-1 receptor antagonist [11]. Increasing the molecular depth of temporal modelling of progression to diabetes before diagnosis may be key to understanding the aetiology of diabetes. While type 1 diabetes and type 2 diabetes are often viewed as two distinct diseases, some individuals exhibit phenotypic features of both diseases, including autoimmunity and insulin resistance [17, 18]. Moreover, the immune system has been shown to drive insulin resistance in type 2 diabetes [15, 16]. It has therefore been suggested that diabetes should be viewed as a continuum [19]. By including both diseases in cohort studies of diabetes, we can improve our understanding of the shared and distinct molecular features of type 1 and type 2 diabetes.

In this study, we analysed plasma samples from 683 blood donors in the nationwide Danish Blood Donor Study (DBDS) biobank to characterise temporal changes in the years leading to a diabetes diagnosis, and to determine the predictive value of such biomarkers together with commonly used risk factors for diabetes.

## Methods

Further details of the methods are given in electronic supplementary material (ESM) Methods.

### Ethics approvals

The study was approved by the National Committee on Health Research Ethics (NVK 1700407) and the Danish Data Protection Agency (P-2019-99).

### Study design and cohort characteristics

In this retrospective case–control study nested within the DBDS, blood samples collected as part of standard blood donations were selected based on the presence or absence of an incident diabetes mellitus diagnosis in the period 2 January 2006 to 31 December 2016. To identify individuals with incident diabetes (non-childhood-onset type 1 diabetes or type 2 diabetes), we created a diabetes register for the DBDS using a modified version of the algorithm described by Carstensen et al [20] covering the period 1977–2016. An initial cohort was constructed based on donation records for all Danish blood donors in the period 2006–2016 (ESM Fig. 1). Individuals were required to fulfil the following criteria: (1) provide valid DBDS consent; (2) have imputed genotype data available; and (3) have had a sample collected as part of inclusion in the DBDS, and at least two other samples at least 9 months apart within the study period. Restriction to individuals for whom imputed genotype data were available resulted in selection of individuals with European ancestry. Based on the above criteria, 344 individuals were identified as having incident diabetes. We randomly sampled 372 individuals without diabetes from 71,095 eligible individuals with a comparable birth year and sex distribution as the individuals with incident diabetes (corresponding to a 1:1.1 sampling ratio), with subsequent removal of six individuals with diabetes. After checking for sample availability in the biobank, 324 individuals with incident diabetes and 359 individuals without diabetes were included in the study. Three consecutive samples obtained at least 9 months apart were analysed for 659 individuals, and two samples were analysed for 24 individuals. We defined the end of follow-up as the date of diabetes diagnosis according to the diabetes register for individuals with incident diabetes. For individuals without diabetes, the end of the follow-up was defined as 31 December 2016, i.e. the end of the available version of the diabetes register. Because of the selection criteria, the cohort is not comparable to the overall DBDS cohort with regard to age and sex distributions, although the regional distribution was similar in the two cohorts (ESM Table 1). As the majority of those in the DBDS cohort are genetically of European ancestry, our cohort is largely representative of the ethnic makeup of the DBDS cohort [21]. Data regarding socioeconomic factors were not available for this study; socioeconomic factors in the DBDS cohort have been described previously [22].

### Biomarker measurements

We measured the concentration of 54 proteins using the V-PLEX metabolic panel 1 human kit and the V-PLEX human biomarker 54-plex kit (Meso Scale Diagnostics, USA) for 2025 samples (ESM Table 2). Additionally, 249 metabolites and lipoprotein particles were analysed for 1866 samples by Nightingale Health (Finland) using targeted metabolomics, of which 171 metabolites in 1863 samples were successfully analysed (ESM Table 2). Polygenic risk scores (PRSs) for 28 phenotypes were calculated using the LDpred2 algorithm [23] (ESM Table 3).

### Phenotype and technical covariates

Sex, date of birth, parental disease history and medication use were extracted from the Danish national registers. Self-reported height, weight and smoking status were extracted from questionnaires completed as part of the DBDS. Data for region, seasonality, time of day when sample was obtained and storage duration were available through the blood bank database.

### Statistical analysis and imputation of missing values

All statistical analyses were performed using R version 4.0.0 (https://www.R-project.org/). Missingness across variables varied between 0.1% and 15% (Table 1 and ESM Table 2). To avoid bias and loss of power, missing values for height, weight, smoking status, time of day when sample was obtained, and protein and metabolite data were imputed using multiple imputations by chained equations as implemented in the mice package (version 3.14.0, https://cran.r-project.org/package=mice) [24].

**Table 1:** Cohort characteristics

Characteristic	Incident DM (*n*=324)	No DM (*n*=359)	Missing values^a^
Sex			
Male	228 (70.4)	270 (75.2)	
Female	96 (29.6)	89 (24.8)	
Age, years			
Baseline	47.9 (40.8–54.4)	49.3 (43.0–55.5)	
End of follow-up	53.5 (45.7–59.3)	56.0 (50.0–62.0)	
Follow-up, years	5.2 (3.9–6.5)	6.7 (4.8–7.8)	
BMI, kg/m^2^	29.8 (26.6–33.2)	25.4 (23.5–28.1)	21, 9
Ever smoked (yes)	159 (51.8)	165 (47.1)	17, 9
Diabetes type			
T1DM	23 (7.1)		
T2DM	301 (92.9)		
Family history of DM^b^			
0	196 (60.5)	298 (83.0)	
1	114 (35.2)	56 (15.6)	
2	14 (4.3)	5 (1.4)	

Values are *n* (%) for binary or categorical data and median (IQR) for continuous variables

^a^The numbers of missing values for BMI and smoking that were imputed are shown as incident DM, no DM

^b^Family history of DM is indicated as the number of parents with DM

DM, diabetes mellitus; T1DM, type 1 diabetes mellitus; T2DM, type 2 diabetes mellitus

The association between the exposures (diabetes diagnosis and diabetes type) and protein and metabolite concentrations was estimated using linear mixed-effects models. This cause–effect relationship between diabetes and systemic biomarker concentrations relies on the assumption that initiation of the pathological cascade and subsequent onset of diabetes occur years before diagnosis [14, 25]. Each biomarker was fitted using diabetes diagnosis (true/false) or diabetes type (type 2 diabetes/type 1 diabetes/no diabetes) as the exposure with person-specific random intercept and slope using the lmer function from the lme4 package (version 1.1-30, https://cran.r-project.org/package=lme4). For each biomarker, two models were fitted. Model 1 was an additive model used to assess the main effect of the diabetes exposure on the biomarker. In model 2, we modelled the time-dependent interaction of diabetes diagnosis for individuals with incident diabetes using restricted cubic splines on time to end of follow-up. For individuals without incident diabetes, the end of follow-up is simply a time point in a normal life course, and the time-dependent interaction was therefore modelled using a linear term on time to end of follow-up. We estimated the per-year effects using estimated marginal means as implemented in the emmeans package (version 1.7.5, https://cran.r-project.org/package=emmeans). Depending on the model fit, models were fitted using either log-transformed or untransformed biomarker concentrations. Estimates and confidence intervals are reported as the relative fold change between individuals with and without incident diabetes. We removed biomarkers for which the model check was not satisfactory from the main results; the effect estimates for these are shown in ESM Table 4.

We used linear methods (Poisson regression with bootstrapping, ‘boot-Poisson’) and non-linear methods (survival random forest model, ‘surv-RF’) to rank the biomarkers according to their predictive value for diabetes diagnosis. Biomarker panels were created based on the rankings from the two methods. For each method, 41 panels were created. Panel 0 serves as a base model consisting only of the covariates age, sex, BMI, smoking status, parental history of diabetes, region, time of day when sample was obtained and seasonality. Panels 1–40 include the top 40 variables ranked based on feature importance, where panel 1 includes covariates and the top biomarker, panel 2 includes covariates and the top two biomarkers, etc., and panel 40 includes covariates and the top 40 biomarkers.

To assess the time-dependent predictive value of the biomarker panels identified above, we assessed the effect of biomarkers on the time-dependent risk of a diabetes diagnosis. We performed Poisson regression analysis using the glm function from the stats R package with log(time to end of follow-up) as an offset. Each model was run on 500 bootstrapped datasets containing approximately two-thirds of the individuals with available data across biomarker data types. All models were assessed for prediction accuracy and calibration using 3-, 5- and 10-year areas under the receiver operating characteristic curve (AUROC), Brier score and Matthew's correlation coefficient (MCC, cumulative risk cut-off = 0.5) for the first (earliest) sample using the one-third holdout sample. The three metrics were summarised as median (2.5th percentile, 97.5th percentile). To account for the unequal distribution of individuals with incident diabetes and individuals without diabetes, each holdout sample was down-sampled to a 50:50 case–control ratio with an equal number of participants in each sample.

All *p* values were adjusted using a false discovery rate (FDR) of 5% for diabetes diagnosis and diabetes type separately.

## Results

We identified 324 blood donors who developed diabetes within the study period from 2006 to 2016 (incident diabetes group) and 359 blood donors without diabetes (non-diabetes group). As expected, individuals with incident diabetes had higher median BMI at baseline and a parental history of diabetes was more common than among individuals without diabetes (Table 1). The majority of individuals with incident diabetes (*n*=301, 93%) were identified as having type 2 diabetes, while 23 individuals (7%) were identified as having non-childhood-onset type 1 diabetes.

**Fig. 1:**
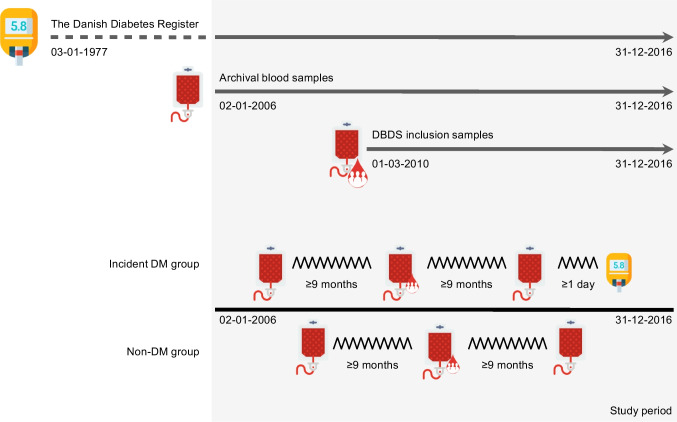
Graphical representation of study set-up. Individuals with incident diabetes were selected based on a diabetes register capturing patients with type 1 or type 2 diabetes (DM) in the period 1977–2016. Only individuals with at least one DBDS inclusion sample collected in the period 2010–2016 and two additional plasma samples (archival sample or DBDS inclusion sample) collected in the period 2006–2016 were included as participants. The three plasma samples had to be donated at least 9 months apart to ensure ample time for molecular changes reflecting the development of diabetes to take place

**Fig. 2:**
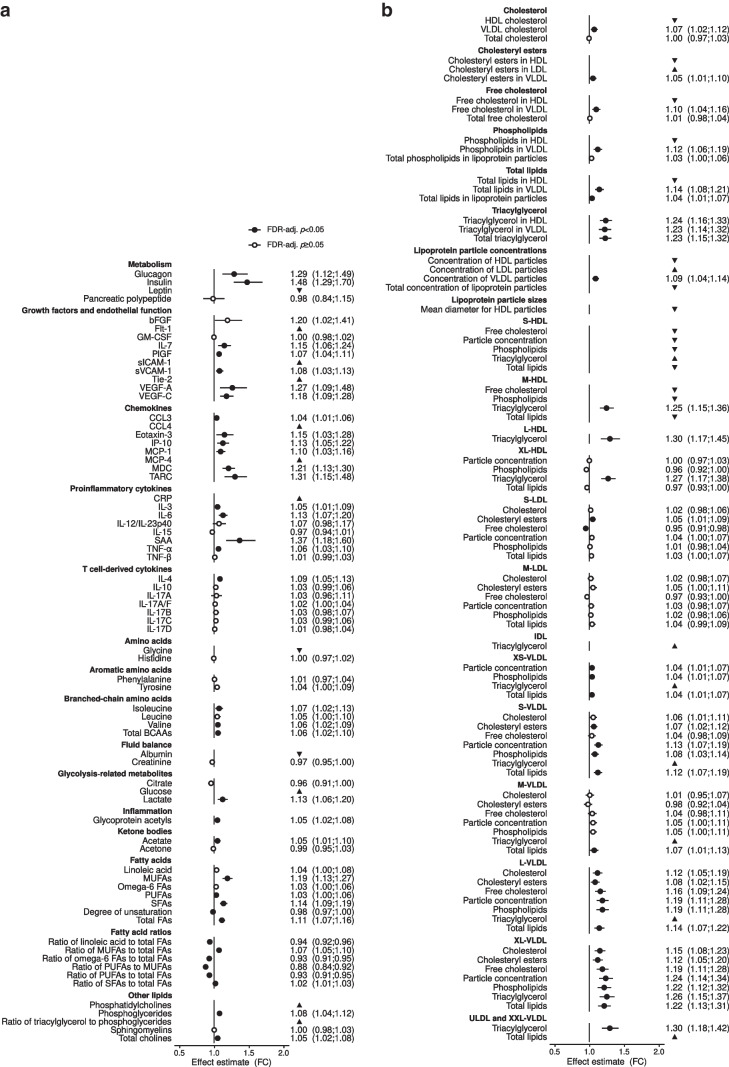
Biomarker-specific effect estimates for incident diabetes. Effect estimates obtained using mixed-effects models (fold change, FC) are shown for proteins (**a**), metabolites (**a**, **b**) and lipoprotein particles (**b**). All estimates are shown as point estimates and 95% CIs. Biomarkers with a significant interaction term are indicated by triangles that show the direction of the trend. Significant associations (FDR-adjusted *p*<0.05) are indicated by filled circles; non-significant estimates are indicated by open circles. bFGF, basic fibroblast growth factor; FA, fatty acids; GM-CSF, granulocyte macrophage colony-stimulating factor; IDL, intermediate-density lipoprotein; IP-10, interferon-induced protein 10; MDC, macrophage-derived chemokine; MUFAs, mono-unsaturated fatty acids; PlGF, placental growth factor; PUFAs, poly-unsaturated fatty acids; SFAs, saturated fatty acids; sVCAM-1, soluble vascular cell adhesion molecule-1; ULDL, ultra low-density lipoprotein; VEGF, vascular endothelial growth factor. The prefixes XS, S, M, L, XL and XXL refer to lipoprotein sizes from extra small to extremely large

We measured the concentration of 225 biomarkers (54 proteins, 73 metabolites and 98 lipoprotein particles) in three plasma samples from 659 individuals and two plasma samples from 24 individuals, obtained during a follow-up period of up to 11 years (Fig. 1, Table 2 and ESM Table 2). The majority of samples were obtained between 0 and 8 years before the end of follow-up (ESM Fig. 2). We identified two proteins, nine metabolites and seven lipoprotein particles that showed a significant temporal relationship with diabetes diagnosis, and 18 proteins, 29 metabolites and 27 lipoprotein particles with an absolute difference between the two groups (Fig. 2 and ESM Table 4). Moreover, we found 21 biomarkers that were significantly different between individuals who developed type 1 or type 2 diabetes (ESM Fig. 3).

**Table 2:** Sample characteristics

Characteristic	Incident DM (*n*=958)	No DM (*n*=1067)	Missing values^a^
Number of samples per participant			
2	14 (4.3)	10 (2.8)	
3	310 (95.7)	349 (97.2)	
Administrative region			
Region H	563 (58.8)	434 (40.7)	
Region M	268 (28.0)	334 (31.3)	
Region N	55 (5.7)	161 (15.1)	
Region SJ	72 (7.5)	138 (12.9)	
Season			
Winter	239 (24.9)	258 (24.2)	
Spring	260 (27.1)	292 (27.4)	
Summer	230 (24.0)	284 (26.6)	
Autumn	229 (23.9)	233 (21.8)	
Time of day when sample was obtained	12:00 (11:00–15:00)	12:00 (10:00–15:00)	151, 144
Storage time, years	11.1 (9.5–12.5)	9.6 (7.7–11.4)	
Sample type			
Archival samples	674 (70.4)	745 (69.8)	
DBDS inclusion samples	284 (29.6)	322 (30.2)	
Medication use			
Statins (ATC code C10)	7 (0.7)	12 (1.1)	
Diuretics (ATC codes C03A and C03C)	6 (0.6)	0 (0.0)	
Immune-modulating (ATC codes H02 and M01)	48 (5.0)	35 (3.3)	

Values are *n* (%) for binary or categorical data and median (IQR) for continuous variables

^a^The numbers of missing values for time of day that were imputed are shown as incident DM, no DM

ATC, anatomical therapeutic chemical classification system; DM, diabetes mellitus

### Markers of glucose metabolism

**Fig. 3:**
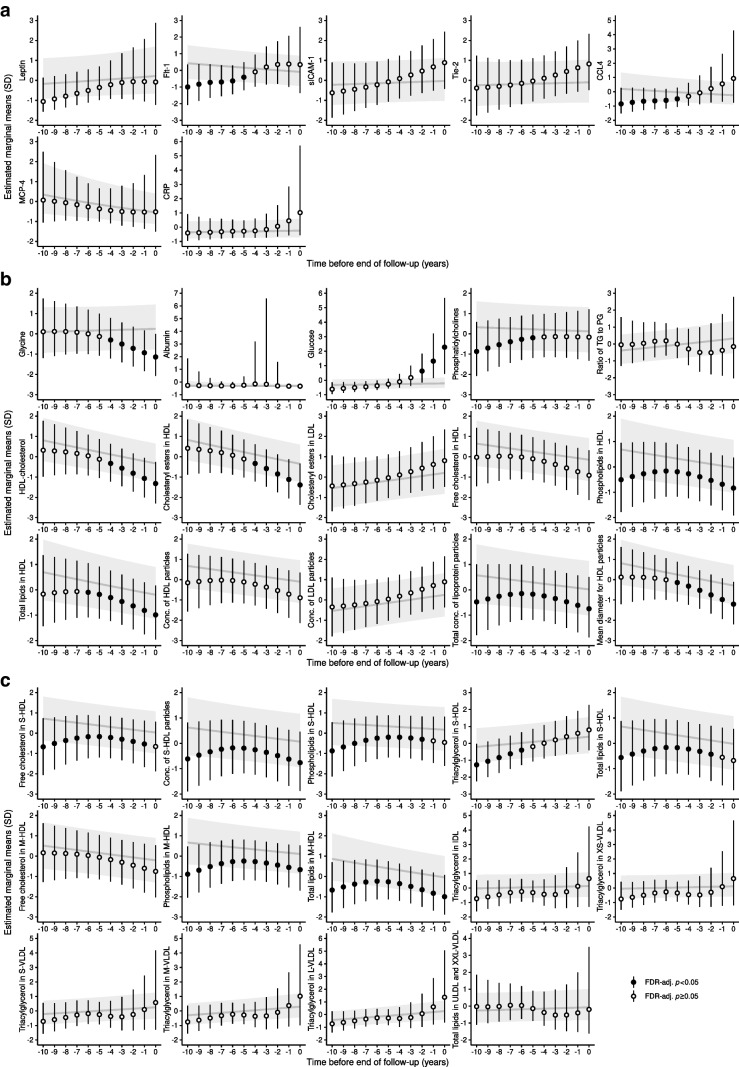
Per-year estimated marginal means for temporally changing biomarkers. Estimated marginal means are shown for proteins (**a**), metabolites (**b**) and lipoprotein particles (**c**) that showed a significant interaction between incident diabetes and time to end of follow-up (FDR-adjusted *p*<0.05) as assessed by ANOVA. Estimated marginal means are shown as point estimates and 95% CIs for the diabetes group (points with error bars) and the non-diabetes group (line with shaded area) for each year before the end of follow-up, i.e. time 0 (diabetes diagnosis for incident diabetes cases and the end of the study period for individuals without diabetes). Values have been *z* score-normalised to ease visualisation; hence one unit difference corresponds to one SD. The exact estimated marginal means are given in ESM Table 4. Significant estimates (FDR-adjusted *p*<0.05) are indicated by filled circles; non-significant estimates are indicated by open circles. Conc., concentration; IDL, intermediate-density lipoprotein; PG, phosphoglycerides; TG, triacylglycerol; ULDL, ultra low-density lipoprotein. The prefixes XS, S, M, L, XL and XXL refer to lipoprotein sizes from extra small to extremely large

We found that plasma glucose increases progressively towards the time of diabetes diagnosis, starting 2 years before diagnosis (Fig. 3a). Insulin and glucagon were 48% and 29% higher, respectively, in the incident diabetes group compared with the non-diabetes group; however, we found no temporal trends for these biomarkers (Fig. 2a). It should be noted that the model check plots for glucose and glucagon did not show an entirely satisfactory model fit, and the estimates should be regarded with caution. In the sub-analysis for diabetes type, we found that glucose levels were significantly lower in the type 1 diabetes group compared with the type 2 diabetes and non-diabetes groups between 5 and 10 years before diagnosis. At 2 years before diagnosis, plasma glucose was significantly higher in the type 1 diabetes group compared with the non-diabetes group, with considerably higher effect estimates than the type 2 diabetes group, although the difference was not significant (ESM Fig. 4). The type 2 diabetes group displayed a similar pattern for plasma glucose as the full diabetes group, with increasing plasma glucose levels at 3 years before diagnosis. Insulin and glucagon levels were lower in the type 1 diabetes group compared with the type 2 diabetes group, and similar to those in the non-diabetes group (ESM Fig. 3).

### Growth factors and markers of endothelial function

Of the ten growth factors and biomarkers of endothelial function that were included, five were higher in the incident diabetes group (Fig. 2a). The levels of a further three markers, soluble intercellular adhesion molecule-1 (sICAM-1), tyrosine protein kinase receptor 2 (Tie-2) and fms-related receptor tyrosine kinase 1 (Flt-1), were found to change towards the time of diabetes diagnosis, although the per-year effects for sICAM-1 and Tie-2 were not significant after FDR adjustment of *p* values (Fig. 3a). sICAM-1 increased 2 years before diagnosis from a relative fold change of 7% (95% CI 1, 14%; *p*_adj_=0.101) to 12% at year zero (95% CI 2, 23%; *p*_adj_=0.101) and Tie-2 increased 3 years before diagnosis from 4% (95% CI 0, 9%; *p*_adj_=0.076) to 10% at year 0 (95% CI 2, 19%; *p*_adj_=0.061) (Fig. 3a). Moreover, we found 20% and 8% lower concentrations of Flt-1, a soluble vascular endothelial growth factor receptor, at 10 and 5 years before diagnosis, respectively, after which it normalised to that of the non-diabetes group (Fig. 3a). When comparing the two diabetes types, Tie-2 showed higher point estimates for the type 1 diabetes group compared with the type 2 diabetes group, Flt-1 showed opposite trends between the two diabetes types, and sICAM-1 showed no time dependency and was only found to be increased in the type 2 diabetes group (ESM Figs 3 and 4).

### Markers of inflammation

Six chemokines, four proinflammatory cytokines and one T cell-derived cytokine showed higher concentrations in the incident diabetes group (Fig. 2a). The largest increases were found for serum amyloid A (SAA) (37%, 95% CI 18, 60%; *p*_adj_=2.21×10^−4^) and thymus and activation-regulated chemokine (TARC) (31%, 95% CI 15, 48%; *p*_adj_=1.03×10^−4^). Moreover, we found that glycoprotein acetyls, another indicator of inflammation, were higher in the incident diabetes group (5%, 95% CI 2, 8%; *p*_adj_=4.97×10^−4^) (Fig. 2a). We found that the chemokine C-C motif chemokine ligand 4 (CCL4) increased towards the time of diabetes diagnosis, starting from a lower level than the non-diabetes group of −44% (95% CI −54, −5%; *p*_adj_=0.049) at 10 years before diagnosis, to −19% at 5 years before diagnosis (95% CI −30, −6%; *p*_adj_=0.049), after which the diabetes group did not differ from the non-diabetes group (Fig. 3a). In the sub-analysis for diabetes type, we found that an inflammatory environment was generally present in both diabetes types, although the point estimates were generally lower for the type 1 diabetes group than the type 2 diabetes group, except for human monocyte chemoattractant protein 4 (MCP-4), which had a point estimate of 54% (95% CI 14, 110%) for type 1 diabetes vs 35% (95% CI 16, 57%) for type 2 diabetes (ESM Fig. 3). Lastly, we found that the IL-17A/F heterodimer was a type 1 diabetes-specific temporal marker, increasing from −61% (95% CI −77, −56%; *p*_adj_=9.30×10^−4^) at 10 years before diabetes diagnosis to 103% (95% CI 26, 226%, *p*_adj_=0.007) at year 0, relative to the type 2 diabetes group, with similar estimates relative to the non-diabetes group (ESM Fig. 4a).

### Amino acids and lipoproteins

We found that the two branched-chain amino acids (BCAAs), isoleucine and valine, as well as total BCAAs, were higher in the incident diabetes group (Fig. 2a), while glycine decreased towards the time of diabetes diagnosis, starting 4 years before diagnosis, with a 21% lower concentration in the year of diagnosis (95% CI −30, −12%; *p*_adj_=1.96×10^−4^) (Fig. 3b). We observed a general trend of higher concentrations of VLDL particles of all sizes and the components of VLDL particles (Fig. 2b), and a significant temporal relationship was observed for HDL-cholesterol and multiple HDL particle features from 10 to 5 years before diagnosis, thereafter decreasing towards the time of diagnosis (Fig. 3b). This trend in non-esterified cholesterol, particle concentration, phospholipids and total lipids appeared to be driven by small HDL (S-HDL) and medium HDL (M-HDL) particles (Fig. 3c). In the sub-analysis for diabetes type, we found no significant differences in isoleucine or glycine between any of the groups, while valine and total BCAAs were only significantly higher in the type 2 diabetes group (ESM Fig. 3). Differences in lipoproteins were only observed for the incident type 2 diabetes cases, while the type 1 diabetes group did not differ from the non-diabetes group (ESM Fig. 3).

### Ranking of biomarkers for time-to-event prediction

**Fig. 4:**
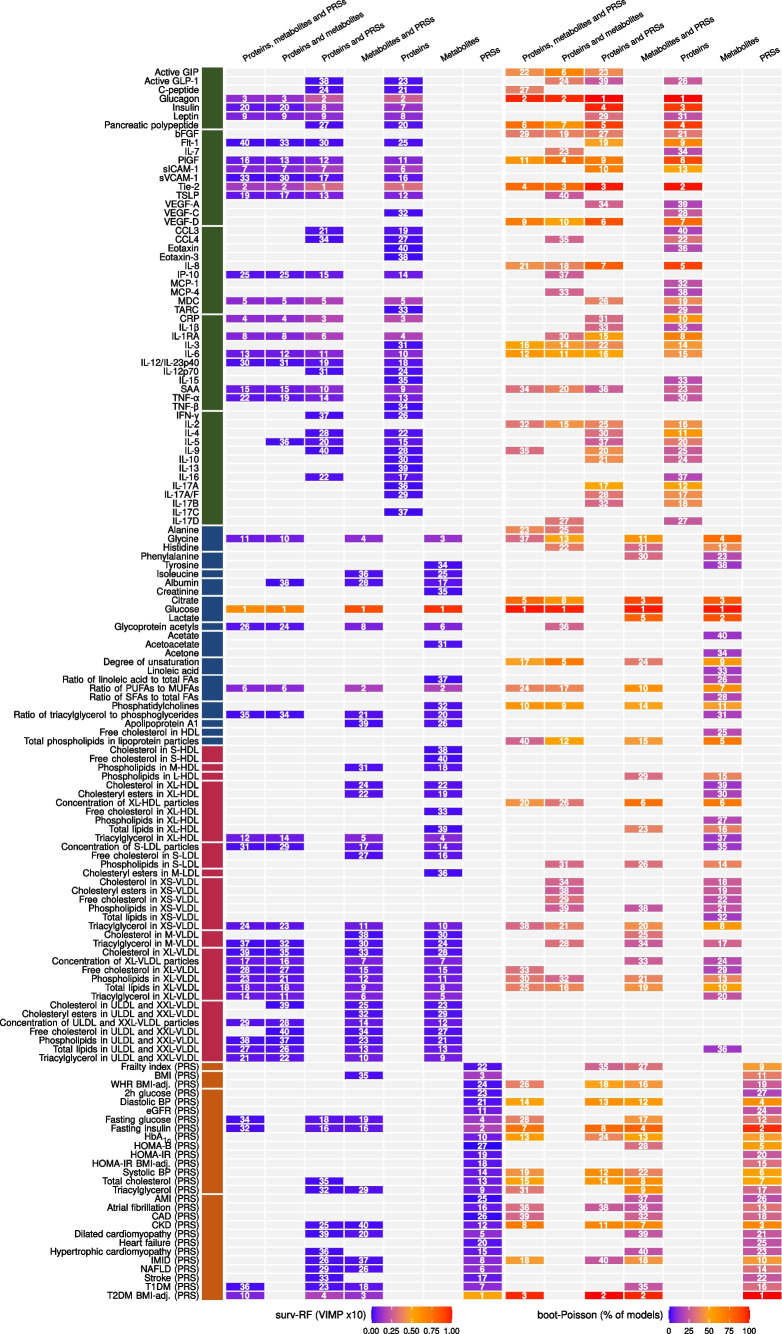
Parameter importance for the top 40 markers from each molecular dataset. Parameter importance for the top 40 markers for each molecular dataset as assessed by variable importance values from the surv-RF model using 100 trees over 1000 bootstraps and the percentage of models where *p*<0.1 for the marker estimate calculated from the boot-Poisson model with 1000 bootstraps. The rank within each combination of biomarker data types is shown in the heatmap. Markers are arranged according to molecular type and groups. Groups are coloured to assist distinction between marker groups. Variable importance has been multiplied by 10 to give a range of 0–1. AMI, acute myocardial infarction; bFGF, basic fibroblast growth factor; CAD, coronary artery disease; CKD, chronic kidney disease; FA, fatty acids; GIP, gastric inhibitory polypeptide; GLP-1, glucagon-like peptide-1; IMID, immune-mediated inflammatory diseases; IP-10, interferon-induced protein 10; MDC, macrophage-derived chemokine; MUFAs, mono-unsaturated fatty acids; NAFLD, non-alcoholic fatty liver disease; PlGF, placental growth factor; PUFAs, poly-unsaturated fatty acids; SFAs, saturated fatty acids; T1DM, type 1 diabetes; T2DM, type 2 diabetes; TSLP, thymic stromal lymphopoietin; ULDL, ultra low-density lipoprotein; VEGF, vascular endothelial growth factor. The prefixes XS, S, M, L, XL and XXL refer to lipoprotein sizes from extra small to extremely large

To rank the measured biomarkers according to their predictive potential in time-to-event models, we used Poisson regression with bootstrapping (‘boot-Poisson’) and survival random forest models (‘surv-RF’). In addition to proteins, metabolites and lipoproteins, we included PRSs for type 1 diabetes, type 2 diabetes and 26 health-related phenotypes (ESM Table 3). We created panels based on the top 40 biomarkers from each biomarker data type (28 for the PRS data) and four combinations of biomarker data types (Fig. 4).

When comparing the 40 top-ranked biomarkers from the 14 model combinations, a few observations stood out. First, plasma glucose had the highest rank in all models where it was included, with the percentage of models ranging from 97 to 100% in the boot-Poisson model and the variable importance ranging from 0.063 to 0.095 in the surv-RF model, which was approximately 4–6 times higher than the variable importance for the second highest marker in any panel (ESM Table 5). Second, a large proportion of the biomarkers identified above as affected by an incident diabetes diagnosis, either temporally or persistently, were included in the panels. For example, the surv-RF 40 top-ranked biomarkers included plasma glucose, Tie-2, CRP, sICAM-1 and glycine, which were all identified as temporally changing biomarkers (Fig. 3). Moreover, the model included IL-1 receptor agonist, which also increases towards the time of diabetes diagnosis but was excluded from the results above due to poor model fit (ESM Table 4). All six biomarkers were found in the top 12 highest ranks. Of the remaining 34 markers, 22 were found to be significantly persistently higher in the diabetes group compared with the non-diabetes group. Only two non-significant markers had a rank of 28 or higher. Surprisingly, the boot-Poisson top 40 markers only included 16 markers that were shown to be significantly different between the diabetes and non-diabetes groups, four of which temporally changed towards the time of diabetes diagnosis, namely plasma glucose, glycine, Tie-2 and phosphatidylcholines. Moreover, in contrast to the surv-RF method, we did not find a clustering of non-significant markers within low ranks. Third, boot-Poisson panels generally included more PRSs and markers with lower concentrations in the diabetes group than the non-diabetes group, while surv-RF gave higher ranks to proteins and VLDL particles (Fig. 4).

### Prediction of diabetes diagnosis

To test the predictive capability of each panel, we modelled the risk of developing diabetes within 3, 5 or 10 years (ESM Fig. 5). For both feature selection methods, the best prediction based on AUROC using all three data types over a 10-year prediction period included 12 biomarkers, both with an AUROC of 0.84 (2.5th, 97.5th percentiles 0.66, 0.96) (ESM Table 6). Addition of 11 biomarkers yielded an increase in AUROC of 0.05 compared with covariates plus plasma glucose (AUROC = 0.79; 0.55, 0.93), a comparable increase to that observed for addition of plasma glucose to the model that only included covariates (AUROC = 0.75; 0.54, 0.91) (ESM Table 7). Boot-Poisson yielded the highest AUROC for 3- and 5-year prediction periods using panel 21 (AUROC = 0.76; 0.56, 0.92) and panel 38 (AUROC = 0.82; 0.62, 0.95), respectively (ESM Table 6).

## Discussion

Diagnostic biomarkers, primarily HbA_1c_ and fasting glucose, are widely used to identify prediabetic and diabetic individuals [26]. However, their benefit is limited for individuals with prediabetes or early stages of type 2 diabetes [26, 27]. Insulin resistance develops years before changes in fasting glucose levels occur [14], indicating that the current clinical biomarkers do not capture early changes in glucose metabolism. Consequently, there is a need to identify novel biomarkers that provide accurate risk estimates for type 2 diabetes at an early, sub-clinical stage to enable early screening and prevention [4, 5].

To our knowledge, this study is the first to describe the temporal trajectories of growth factors and markers associated with endothelial dysregulation prior to a diabetes diagnosis, although these biomarkers are well known to be associated with microvascular diabetes complications such as retinopathy [28–30] and nephropathy [31]. We found that all but two of the measured markers were higher in the diabetes group or increased towards the time of diabetes diagnosis. The majority of these were found to be significantly increased for both types of diabetes. We found that Flt-1, sICAM-1 and Tie-2 increased towards the time of diabetes diagnosis, although the per-year effects were not significant for sICAM-1 and Tie-2 after FDR adjustment. Flt-1 has been found in higher concentrations in individuals with type 2 diabetes, but not in individuals with impaired fasting glucose [32]. sICAM-1 has been found to be associated with the development of both retinopathy [33] and nephropathy [34], and Tie-2 has been shown to be associated with nephropathy [35], but may have a protective role in retinopathy [36]. Both sICAM-1 and Tie-2 were found to increase approximately 2–3 years before diabetes diagnosis. This is in line with previous studies showing that nephropathy and retinopathy are some of the earliest clinical indications of diabetes [37, 38].

We identified panels of 12 biomarkers that improved the prediction of diabetes up to 10 years before a diagnosis compared with a base model that comprised commonly used risk factors (age, BMI, parental history of diabetes and plasma glucose). The best-performing panels included all biomarker data types, i.e. PRSs, proteins and metabolites. The inclusion of biomarkers to assess diabetes risk has the potential to improve screening, which has been shown to have only moderate utility when based on HbA_1c_ alone [39]. Our findings may be used to develop new risk assessment tools for early detection of the development of diabetes using a single non-fasting plasma sample.

Our analysis showed impaired glucose metabolism and dyslipidaemia in the incident diabetes group. The incident diabetes group had increasing plasma glucose, decreasing glycine and elevated insulin and glucagon levels. Glycine is a known marker of insulin sensitivity [40], and it is therefore noteworthy that the change in glycine levels precedes the increase in plasma glucose. Moreover, the incident diabetes group had a high concentration of VLDL particles, decreasing concentrations of HDL particles towards the time of diabetes diagnosis (driven by S-HDL and M-HDL particles), and high triacylglycerol concentrations in all lipoprotein particles. This pattern was exclusive to the type 2 diabetes group, in agreement with previous reports [41, 42]. Moreover, as previously reported for insulin resistance [43] and incident type 2 diabetes [44], HDL particle size decreased towards the time of diabetes diagnosis. The decrease in HDL particles and size occurred concurrently with the decrease in glycine, indicating that HDL-related dyslipidaemia and insulin resistance develop simultaneously in type 2 diabetes.

While type 1 and type 2 diabetes are characterised by distinct pathologies, phenotypic features such as autoimmunity and insulin resistance are seen in both patient populations [17, 18]. Moreover, diseases such as retinopathy, nephropathy and neuropathy are comorbid with both diseases, probably due to the damaging effects of prolonged hyperglycaemia [45, 46]. In this study, we estimated the associations between 225 biomarkers and type 1 diabetes plus type 2 diabetes together in the main analysis, and separately in a sub-analysis of the two diabetes types. Most individuals with incident diabetes were classified as having type 2 diabetes (93%), and thus the results between the main analysis and the sub-analysis did not differ considerably for this group. However, for the individuals with non-childhood-onset type 1 diabetes (7%), we found a largely similar inflammatory profile, except for a type 1 diabetes-specific increase in IL-17A/F. IL-17A/F, together with IL-17A and IL-17F, has been linked to beta cell pathogenesis in type 1 diabetes and multiple autoimmune diseases through activation of the IL-1 receptor agonist [47, 48].

### Study limitations

There are several limitations in working with historical biobank samples. First, BMI was only recorded by means of questionnaires, and, in most cases, a BMI value was only available for a single point in time. Adjustment for BMI may therefore not be accurate over large time intervals. Second, for some metabolomics measurements, there was considerable sample degradation affecting the measured levels. We therefore only report fold changes between groups of individuals. Moreover, we have chosen not to present the results for biomarkers with poor model fit in the main text, as estimates from these models may not accurately represent underlying biological differences. Third, as our cohort consists of blood donors with a minimum age of 18 years, all individuals with type 1 diabetes were diagnosed in adulthood. Therefore, our results reflect the molecular changes occurring in adult-onset type 1 diabetes and may not translate to childhood-onset type 1 diabetes. Moreover, as our cohort only includes 23 individuals with type 1 diabetes, we have limited power to detect type 1 diabetes-specific effects and model these temporally. Fourth, as only a few samples were available for the very early time points (more than 7–8 years before the end of follow-up), the estimates for these time points are underpowered and should be considered with caution (ESM Fig. 2). Moreover, as the diagnosis date for individuals with diabetes is based on registry data, it does not necessarily reflect the exact time of diabetes onset. Fifth, as the DBDS has strict criteria for inclusion, the cohort may be affected by the healthy donor effect, for example [49]. Hence, some caution should be exercised when translating the presented results to a general, non-donor population.

### Conclusion

Our study greatly expands the number of biomarkers that are known to temporally change in the progression of diabetes before diagnosis. The identified biomarkers align with the known pathologies of type 1 diabetes and type 2 diabetes, and show both shared and distinct molecular patterns between the two diabetes types. Our findings are useful for understanding the sequence of pathological changes that occur in the development of diabetes. Moreover, our findings may, upon independent replication, be used to identify clinical biomarkers for the early detection of individuals with an increased risk of developing diabetes.

## Supplementary Information

Below is the link to the electronic supplementary material.

Supplementary file1 (PDF 1515 KB)

Supplementary file2 (XLSX 546 KB)

## Data Availability

Data cannot be publicly shared but may be made available upon request. Requests should be directed to the corresponding author. Approval is contingent on adherence to Danish law and may be subject to restrictions.

## Abbreviations

AUROC: Area under the receiver operating curve
BCAA: Branched-chain amino acid
CCL4: C-C motif chemokine ligand 4
CRP: C-reactive protein
DBDS: Danish Blood Donor Study
FDR: False discovery rate
Flt-1: fms-related receptor tyrosine kinase 1
MCP-4: Monocyte chemoattractant protein 4
M-HDL: Medium HDL
PRS: Polygenic risk score
RF: Random forest
SAA: Serum amyloid A
S-HDL: Small HDL
sICAM-1: Soluble intercellular adhesion molecule-1
TARC: Thymus and activation-regulated chemokine
Tie-2: Tyrosine protein kinase receptor 2
