# Supplementary material for: Longitudinal metabolite and protein trajectories prior to diabetes mellitus diagnosis in Danish blood donors: a nested case–control study
Source: Diabetologia. 2024 Jul 30;67(10):2289–303. doi: 10.1007/s00125-024-06231-3 (PMC11446992; doi:10.1007/s00125-024-06231-3)
Supplement: Supplementary file 1 — Supplementary file1 (PDF 1515 KB) [file 125_2024_6231_MOESM1_ESM.pdf]

ELECTRONIC SUPPLEMENTARY MATERIALS

Longitudinal metabolite and protein trajectories prior to diabetes mellitus diagnosis in Danish blood donors

Agnete T. Lundgaard[1], David Westergaard[1,2,3], Timo Röder[1], Kristoffer S. Burgdorf[1], Margit H. Larsen[4], Michael Schwinn[4], Lise W. Thørner[4], Erik Sørensen[4], DBDS Genomic Consortium, Kaspar R. Nielsen[5], Henrik Hjalgrim[6,7,8,9], Christian Erikstrup[10,11], Bertram D. Kjerulff[10,11], Lotte Hindhede[10], Thomas F. Hansen[1,12], Mette Nyegaard[13], Ewan Birney[14], Hreinn Stefansson[15], Kári Stefánsson[15], Ole B.V. Pedersen[16,17], Sisse R. Ostrowski[4,16], Peter Rossing[16,18], Henrik Ullum[19], Laust H. Mortensen[1,2,20], Dorte Vistisen[18,21], Karina Banasik[1], Søren Brunak[1]

1. Novo Nordisk Foundation Center for Protein Research, Faculty of Health and Medical Sciences, University of Copenhagen, Copenhagen, Denmark
2. Methods and Analysis, Statistics Denmark, Copenhagen, Denmark
3. The Recurrent Pregnancy Loss Unit, Copenhagen University Hospitals Rigshospitalet and Hvidovre, Copenhagen, Denmark
4. Department of Clinical Immunology, Copenhagen University Hospital, Rigshospitalet, Copenhagen, Denmark
5. Department of Clinical Immunology, Aalborg University Hospital, Aalborg, Denmark
6. Danish Cancer Society Research Center, Copenhagen, Denmark
7. Department of Epidemiology Research, Statens Serum Institut, Copenhagen, Denmark
8. Department of Haematology, Copenhagen University Hospital, Rigshospitalet
9. Department of Clinical Medicine, Copenhagen University, Copenhagen, Denmark
10. Department of Clinical Immunology, Aarhus University Hospital, Aarhus, Denmark
11. Department of Clinical Medicine, Health, Aarhus University, Aarhus, Denmark
12. Department of Neurology, Copenhagen University Hospital - Rigshospitalet, Glostrup, Denmark
13. Department of Health Science and Technology, Faculty of Medicine, Aalborg University, Aalborg, Denmark
14. European Molecular Biology Laboratory, European Bioinformatics Institute, Cambridge, United Kingdom
15. deCODE Genetics, Reykjavik, Iceland
16. Department of Clinical Medicine, Faculty of Health and Medical Sciences, University of Copenhagen, Copenhagen, Denmark
17. Department of Clinical Immunology, Zealand University Hospital, Køge, Denmark
18. Steno Diabetes Center Copenhagen, Herlev, Denmark
19. Statens Serum Institut, Copenhagen, Denmark
20. Department of Public Health, University of Copenhagen, Copenhagen, Denmark
21. Novo Nordisk A/S, Bagsværd, Denmark

ESM Methods

Ethical approvals

The study was approved by the National Committee on Health Research Ethics (NVK 1700407) and the Danish Data Protection Agency (P-2019-99).

Study design

In this retrospective case-control study nested within the Danish Blood Donor Study (DBDS), blood samples collected as part of standard blood donations were selected based on the presence or absence of an incident diabetes diagnosis in the period 2 January 2006 to 31 December 2016.

An initial cohort of Danish blood donors was constructed based on donation records for all Danish blood donors in the period 2006-2016 (ESM Fig. 1). Individuals were required to fulfil the following criteria: 1) a valid DBDS consent, 2) imputed genotype data available, 3) having a DBDS inclusion sample and at least two other samples at least nine months apart within the study period. Restricting on individuals with imputed genotype data available imposed a selection of individuals with European ancestry. Based on the above three criteria, 344 individuals were identified as having incident diabetes by linking to a DBDS diabetes register (see below). We randomly sampled 372 individuals without diabetes from 71,095 eligible individuals with comparable birth year and sex distribution as the individuals with incident diabetes corresponding to a 1:1.1 sampling ratio and subsequent removal of six individuals with diabetes. After checking for sample availability in the biobank, 324 individuals with incident diabetes and 359 individuals without diabetes were included in the study. Of these, three consecutive samples nine months apart were available for 659 individuals, and two samples were collected for 24 individuals. We defined the end of the follow-up as the date of diabetes diagnosis according to the diabetes register for individuals with incident diabetes. For individuals without diabetes, the end of the follow-up was defined as 31 December 2016, i.e. the end of the available version of the diabetes register.

Due to the selection criteria, the cohort is not comparable to the overall DBDS cohort in regard to age and sex distributions, while the regional distribution was similar in the two cohorts (ESM table 1). As the majority of the DBDS cohort is genetically of European ancestry, our cohort is largely representative of the ethnic makeup of the DBDS cohort [1]. Data regarding socioeconomic factors were not available for this study; Socioeconomic factors in the DBDS cohort have previously been described by others [2].

The DBDS diabetes register

Individuals from DBDS with incident diabetes (type 1 and type 2 diabetes) were identified using a modified version of the algorithm created by Carstensen *et al* [3] to create a DBDS diabetes register

covering the period 1977-2016. In short, information on diabetes-related diagnoses and treatments was compiled from national patient registries in Denmark. Three registries were used: 1) the Danish National Patient Registry (NPR) [4] was used to identify individuals diagnosed with type 1 diabetes, type 2 diabetes, or unspecified diabetes at Danish hospitals in the period 1977-2016); 2) the Danish National Prescription Registry (DNPR) [5] was used to identify individuals who retrieved a prescription for insulins (Anatomical Therapeutic Chemical (ATC) code A10A) or other anti-diabetes drugs (ATC code A10B) in the period 1995-2016); and 3) the Danish National Health Service Register (NHSR) [6] was used to identify individuals who received diabetes-related food treatments through Danish health professionals in the period 1990-2016. To be identified as having diabetes, a person must have either two entries in NPR or DNPR or a single entry in NHSR. The date of diabetes diagnosis was based on the second entry for NPR and DNPR or the first entry in NHSR, whichever came first. Individuals with an indication of polycystic ovary syndrome (PCOS) or gestational diabetes mellitus (GDM) were removed (for details, see [3]). Classification of diabetes type was done as described in [3]. We did not have access to the Danish Adult Diabetes Registry (DADR) or the Danish Eye-Screening Database for diabetes patients included in the Danish National Patient Registry.

As the classification of diabetes type in the original algorithm was in part based on the diabetes type ascribed in DADR, we investigated the distribution of the type 1 diabetes and type 2 diabetes polygenic risk scores (PRSs) (for details see below) for the study participants included in the diabetes register. We found that the type 1 diabetes PRS distribution for individuals classified as having type 1 diabetes was shifted to the right when compared to individuals classified as having type 2 diabetes and individuals without a diabetes diagnosis and that the distributions of individuals classified as having type 2 diabetes almost completely overlapped with the distribution for the individuals without diabetes (ESM Fig. 6a). A similar finding was found for the BMI-adj. type 2 diabetes PRS, albeit to a lesser extent (ESM Fig. 6b).

The Danish Blood Donor Study

The Danish Blood Donor Study (DBDS) is a multi-center cohort study based on the existing blood bank infrastructure in Denmark [7]. Since 2010, blood donors aged 18-67 have been invited to participate in the DBDS. In Denmark, blood donation is voluntary and donors are asked to only donate blood if they “feel generally healthy” at the time of donation. Exclusion criteria with regards to e.g. specific medical conditions and medication use exist [7]. Donors are asked to eat and drink before donation to minimise risks of adverse reactions to the blood draw. For details on the information collected from individuals in DBDS see [1, 7]. Since 2006, it has been standard practice in Danish blood banks to store a plasma sample from all blood donations for testing and research purposes as part of the blood donation. The blood samples are stored at -20°C as EDTA-treated plasma. The Danish healthcare system is managed by five administrative regions. Across these regions, blood bank

procedures differ and DBDS inclusion has varied. In the current study, samples from four of the five regions have been included (Table 2).

Biomarker measurements

From the final cohort of 683 individuals, 2,025 samples were aliquoted and analysed using the V-PLEX Metabolic Panel 1 Human Kit and V-PLEX Human Biomarker 54-Plex kit from Meso Scale Diagnostic. Additionally, 1,866 samples were sent to Nightingale Health for metabolomics analysis, of which 1,863 samples were successfully analysed.

Protein biomarkers were measured using the V-PLEX Metabolic Panel 1 Human Kit and V-PLEX Human Biomarker 54-Plex kit from Meso Scale Diagnostic. Due to the poor quality observed for the TH17 panel, the panel was excluded (unpublished data). Sample measurements were conducted at three different sites: 1) the Department of Clinical Immunology, Aarhus University Hospital, Aarhus, Denmark; 2) the Department of Clinical Immunology, Copenhagen University Hospital, Rigshospitalet, Copenhagen, Denmark, and 3) the Department of Health Technology, the Technical University of Denmark, Lyngby, Denmark. In short, 150 µL EDTA-treated plasma was thawed and aliquoted into 96-well plates on an automated platform (Hamilton MicroLab STAR liquid-handling platform, Reno, USA) at the Department of Clinical Immunology, Copenhagen University Hospital, Rigshospitalet, Copenhagen, Denmark. 96-well plates with the plasma samples were refrozen until the time of analysis. Due to the observed batch effect between plates, raw signal values were preprocessed using batch control procedures. In short, signal values were log2-transformed, outliers were removed based on manual inspection of panel-specific PCA plots, and the values were median-normalised. The effectiveness of batch correction was assessed by PCA plots and density plots. Two biomarkers, IL-8 and VEGF-A, were assayed on two panels. For IL-8, we only included measurements from the Pro1 panel due to poor range coverage on the Chem1 panel. For VEGF-A, we calculated the average of the two panels (Angio1 and Cyto1) after normalisation and used the mean values for downstream analysis. Values were back-transformed before inclusion in linear mixed-effects models (see below).

Metabolomics analysis of 249 analytes was conducted using high-throughput spectroscopy by Nightingale Health (Nightingale Health Plc). Measurements of glutamine and Omega-6/Omega-3 ratios were excluded due to suboptimal sample conditions. Pyruvate measurements were excluded due to interference by EDTA treatment of plasma. 3-Hydroxybyturate measurements were excluded due to ethanol contamination in ~1/3 of the samples. Assays with more than 50% of samples below the limit of detection were removed (Omega-3, DHA, triacylglycerol in S-LDL particles, and triacylglycerol in XL-HDL particles). For all other assays, values for measurements below the limit of detection were assigned by Nightingale Health Plc. Assigned values exacting zero were replaced with values sampled from a uniform distribution ranging from zero to the lowest assigned or observed value above zero.

All lipoprotein proportions were excluded. The full list of included assays, including information on missingness and measurements below the limit of detection can be found in ESM Table 2.

Genotyping and polygenic risk scores

Genotyping and imputation of the DBDS Genetic cohort are described in Hansen *et al* [1]. Polygenic risk scores (PRS) were calculated using the LDpred2 algorithm [8] implemented in the R package bigsnpr (version 1.5.2, https://cran.r-project.org/package=bigsnpr) with R version 3.5.05231. Autosomal genotypes from 90,598 individuals in the DBDS genomic cohort were filtered to only include variants present in the HapMap3 set of 1,120,696 reference variants. Any missing genotype information was replaced with a homozygous reference genotype of the affected locus. We identified a set of 978,246 genotyped variants present in both genotype and GWAS summary statistics data that we subsequently subjected to Ldpred2's recommended standard deviation quality control, which assures that genotype standard deviations are of similar scale in the GWAS data and the genetic cohort of interest. After variant matching and quality control, a mean of 969,607 (S.D. 8,777) variants remained for per-chromosome risk score calculation for each of the 28 traits. We used the LDpred2-auto algorithm with 30 Gibbs sampling chains, 1,000 burn-in iterations, and 500 iterations after burn-in. The initial values for the 30 sampling chains were a) the LDSC regression estimate for heritability h2 (same for all chains); b) one of 30 initial values for the proportion of causal variants p, evenly spaced on a logarithmic scale from 0.0001 to 0.9. Final per-chromosome effect sizes were calculated from each set of 30 sampling chains (per trait and chromosome) through a three-step process, which serves to ensure that the model as a whole (spanning 30 chains) converged: 1) computing the standard deviations of each chains' predicted scores, 2) keeping only the chains within three median absolute deviations from the median standard deviation, 3) averaging the effect sizes of the remaining chains. Across the 616 per-chromosome models (28 traits times 22 chromosomes), 27 chains were included in the final score on average. The lowest number of included chains was 18. Finally, the resulting per-chromosome risk scores were added together into genome-wide PRSs. For information on the summary statistics used to calculate PRSs, see ESM Table 3. All PRSs were subsequently *z* score transformed to enable meaningful reporting of effects.

Phenotypic and technical covariates

To remove residual effects on biomarker levels caused by phenotypic and technical sources, we included information from multiple sources as covariates in our models. Sex, age, BMI, and smoking are known to be associated with the risk of diabetes [3, 9, 10] and can affect biomarker levels [11, 12] making them likely confounding for the biomarker-diabetes associations. Season [13], time of day [14, 15], and the use of certain medications (here we include statins, thiazides and loop diuretics, and immune-modulating drugs) [15–17] can affect a broad spectrum of circulating biomarkers and were therefore included as covariates in the models to reduce noise. Storage time and sample handling (here

region and sample type are used as proxies) can affect protein and metabolite concentrations in plasma samples [13, 18] and were likewise included in the models to reduce noise. We further included parental history of diabetes in the Poisson models for the prediction of diabetes (see below), as family history is an independent risk factor for diabetes [19] and the information is often readily available in a clinical setting.

Sex and date of birth were extracted from the Danish National Central Person Registry (CPR) [20]. Parental history of diabetes was identified using the Danish National Patient Register (NPR) [4] and the DBDS diabetes register (see above). A parent was identified as having diabetes by either having at least one of the four diagnosis codes (The International Classification of Diseases (ICD)-10 E10, E11, E14, and O24.4 (and their corresponding ICD-8 codes)) in NPR spanning the period 1 January 1973 to 13 September 2019 or by being included in the diabetes register. Parental history of diabetes was coded as 0, 1, or 2 depending on the number of parents with a diabetes diagnosis.

Self-reported height, weight, and smoking status were extracted from questionnaires obtained as part of the DBDS. BMI was calculated after MICE imputation (see below) using the formula BMI = weight (kg)/height (m)^2. Smoking status was defined as never smoked or ever smoked (past or current smoker). For the majority of individuals (*n*=492, 72%) a single questionnaire was available and values from this were used for all three sample time points. If multiple questionnaires were available, the information was updated for subsequent time points with the information retrieved closest to the sample used. We imputed the respective values for 30 individuals (4.4%) where neither height, weight, or both were registered in any questionnaire and for 26 individuals (3.8%) where smoking status was missing from the questionnaire.

Information on medication use was extracted from the Danish National Prescription Registry [5]. Prescriptions of statins (ATC code C10), thiazides and loop diuretics (ATC codes C03A and C03C), and immune-modulating drugs (ATC codes H02 and M01) were included to account for lipid decreasing, lipid increasing, and anti-inflammatory/immunosuppressant effects, respectively. We assumed that individuals were under treatment during a blood donation if a prescription had been filled a maximum of six months before and six months after the donation.

Information regarding sample retrieval and storage was obtained through the blood bank database. Donation sites were grouped according to the administrative regions of Denmark (Capital Region (Region H), Mid Jutland (Region M), North (Region N), and Zealand (Region SJ)). Seasonality was calculated using sine and cosine transformation of day and month for each sample donation. The time of day when the sample was obtained was calculated as minutes from midnight. Storage time was

calculated as the time from the date of sample donation to 31 December 2021. Two types of samples, archival and DBDS inclusion samples, were used in this study. The two sample types varied slightly in sampling handling and storage from collection in the blood bank until the time of sample retrieval.

Imputation

Missingness across variables varied between 0.1% and 15% (Table 1 and ESM Table 2). Missing values for height, weight, smoking status, and time of day when sample was obtained were imputed together with all protein markers, technical and phenotype covariates in 50 copies with 5 iterations using Multiple Imputations by Chained Equations (MICE) method [21] (mice package version 3.14.0, https://cran.r-project.org/package=mice) with missing-at-random assumptions, i.e. that missingness was random conditional on covariates. To utilise intra-individual information, 2l.pan was used for continuous variables and 2l.bin for binary variables with random slopes and intercepts for time and individual, respectively. After imputation, all continuous variables were *z* score normalised to unify the value ranges with the exception of the sine and cosine measures of seasonality, as the range of values for these variables lies between 0 and 1. When appropriate, each dataset was independently included in the analysis and estimates were averaged across copies using Rubin's rules [22].

Similarly, a separate imputed dataset was created for feature selection and Poisson modelling including technical and phenotype covariates, all protein markers, and all metabolites and lipoprotein particles for the 1,863 samples with a successful metabolomics analysis. Due to the large number of variables, we used the quickpred function from the MICE package to pre-set the prediction matrix to only include predictors with an absolute Spearman correlation of ≥0.25. Moreover, assays with identical missingness patterns were excluded as predictors for the corresponding target. The imputation was performed as above in 50 copies with 10 iterations.

Linear mixed models

The association between the exposures (diabetes diagnosis and diabetes type) and protein and metabolite concentration was estimated using linear mixed-effects models. This cause-effect relationship between diabetes and systemic biomarker concentrations relies on the assumption that the initiation and onset of diabetes occur years before a diabetes diagnosis [23, 24]. Each biomarker was fitted with diabetes diagnosis (true/false) or diabetes type (type 2 diabetes/type 1 diabetes/no diabetes) as exposure with person-specific random intercept and slope using the lmer function from the lme4 package (version 1.1-30, https://cran.r-project.org/package=lme4). For each biomarker, two models were fitted. Model 1 is an additive model to assess the main effect of diabetes outcome on the biomarker. In model 2, we modelled the time-dependent effect of diabetes diagnosis for individuals with incident diabetes with restricted cubic splines (RCS) on time to the end of follow-up. The model specifications were as follows:

Model 1: *lmer(*$biomarker_i \sim diabetes_{status/type} + time_{end\ of\ follow\text{-}up}$ +

age + age^2 *+ sex + BMI + smoking +*

diuretic use + statin use + anti-inflammatory use +

region + time of day when sample was obtained + sin(month, date) + cos(month, date) +

sample type + storage time + sample type:storage time +

($time_{end\ of\ follow\text{-}up}$*|ID),*

data = data

)

Model 2: *lmer(*$biomarker_i \sim diabetes_{status/type} + time_{end\ of\ follow\text{-}up}$ +

I($diabetes_{status/type}$*=case):rcs(*$time_{end\ of\ follow\text{-}up}$*) +*

I($diabetes_{status/type}$*=control):*$time_{end\ of\ follow\text{-}up}$ +

age + age^2 *+ sex + BMI + smoking +*

diuretic use + statin use + anti-inflammatory use +

region + time of day when sample was obtained + sin(month, date)$_{donation}$ + *cos(month, date)*$_{donation}$ +

sample type$_{donation}$ + *storage time*$_{donation}$ + *sample type*$_{donation}$*:storage time*$_{donation}$ +

($time_{end\ of\ follow\text{-}up}$*|ID),*

data = data

)

Restricted cubic splines (RCS) on time to end of follow-up were modelled using the rms package (version 6.3-0, https://cran.r-project.org/package=rms). We selected the number of knots based on the lowest Akaike information criterion (AIC), using 3 (percentile 0.1, 0.5, 0.9), 4 (percentile 0.05, 0.35, 0.65, 0.95), or 5 (percentile 0.05, 0.275, 0.5, 0.725, 0.95) knots in the RCS [25]. For individuals without diabetes, year zero (end of follow-up) is simply a time point in a normal life course and therefore the time-dependent effects were modelled using a linear term. The inclusion of an interaction term modelling a temporal relationship between biomarker and diabetes diagnosis was based on a significant improvement (FDR-adj. p value < 0.05) in model fit assessed by ANOVA analysis using the Wald method, as implemented in the anova function from the mice package (version 3.14.0, https://cran.r-project.org/package=mice). For biomarkers that had significant interaction between diabetes diagnosis/diabetes type and time to end of follow-up, the effect estimates for each year (-10 to 0) were computed as estimated marginal means (EMMs) using the emmeans and pairs functions from the emmeans package (version 1.7.5, https://cran.r-project.org/package=emmeans). The per-year p values were FDR-adjusted for each biomarker separately.

To account for skewness in biomarker measurements, we performed the modelling both with and without natural log-transformation of the biomarker measurements. The use of log transformation for each biomarker was determined by visual inspection of QQ plots and is reported in ESM Table 4. Effect estimates and confidence intervals are reported as relative differences (fold change) calculated as the difference from the model intercept or by exponentiation, respectively. The models were adjusted for time to end of the follow-up period, age and age^2, sex, BMI, smoking status, sample type (archival sample or DBDS inclusion sample), storage time, region, time of day when sample was obtained, season, statin use, diuretics use (thiazides and loop-diuretics), and anti-inflammatory drug use (corticosteroids and NSAIDs) use, as fixed effects. Model checks were conducted by visual inspections of the QQ plots and residual distributions (histogram of residuals and residuals vs. fitted value). We removed biomarkers for which the model check was not satisfactory from the main results, the effect estimates of which can be found in ESM Table 4.

Feature selection

We employed a linear (boot-Poisson regression with bootstrapping, "boot-Poisson") and a non-linear (survival random forest model, "surv-RF") method to determine variable importance (VIMP) for the prediction of diabetes diagnosis.

boot-Poisson: 1,000 datasets were created using bootstrapping including ~2/3 of individuals and for each sample, a random imputation was chosen. A Poisson model (see below) with all biomarkers and covariates was fitted to each dataset. Across all datasets, the number of models where a biomarker had a p value < 0.1 was counted. The biomarkers were ranked on variable importance according to the model count.

surv-RF: A survival random forest model performed using the rfsrc function from the randomForestSRC package (version 3.1.1, https://cran.r-project.org/package=randomForestSRC) with 100 trees. The model was fitted on 1,000 datasets with one randomly selected single sample per individual. The mean variable importance (VIMP) was calculated across the 1,000 samples and ranked based on the highest mean VIMP.

To reduce collinearity between input features, we removed biomarkers with a Spearman correlation above 0.9 using the Hobohm II algorithm [26] before running both methods (ESM Fig. 7).

For each method, we created 41 panels. Panel 0 serves as a base model consisting of covariates only: age, sex, BMI, smoking status, parental history of diabetes, region, time of day when the sample was obtained, and seasonality. Panel 1-40 includes the top 40 variables ranked based on feature importance, where panel 1 includes covariates and the top one biomarker, panel 2 includes covariates and the top two biomarkers, etc., and panel 40 includes covariates and the top 40 biomarkers. The choice of the number of panels was based on the computational requirements of the Poisson regression (see below), which prevented testing panels containing all biomarkers. Moreover, initial

tests showed very small improvements in prediction metrics beyond the inclusion of 10-20 biomarkers, indicating that the testing of more panels would have limited utility.

Poisson regression of time to diabetes diagnosis

To assess the time-dependent predictive value of the biomarker panels identified above, we assessed the effect of biomarkers on the time-dependent risk of a diabetes diagnosis. We used the glm function from the stats package (R 4.0.0, https://www.R-project.org) with log(time to end of follow-up) as an offset. Each model was run on 500 bootstrapped datasets containing approximately two-thirds of individuals with available data across biomarker data types. In all models, the biomarker measurements were the main predictors. Moreover, we included age, sex, BMI, smoking status, parental history of diabetes, region, time of day when the sample was obtained, and seasonality as covariates. For all covariates but sex and parental history of diabetes, the values were updated for each sample, insofar as the value had changed between samples. All models were assessed using 3-, 5-, and 10-year areas under the receiver operating characteristic curve (AUROC), Brier score, and Matthew's Correlation Coefficient (MCC, cumulative risk cut-off=0.5) for prediction accuracy and calibration for the first (earliest) sample using the one-third holdout sample. The three metrics were summarised as median (2.5th percentile, 97.5th percentile). To account for the unequal distribution of individuals with incident diabetes and individuals without diabetes, each holdout sample was down-sampled to a 50:50 case-control ratio with an equal number of participants in each sample.

Statistical analysis

All analyses were conducted in R version 4.0.0 (https://www.R-project.org/) if not stated otherwise. All p values were adjusted using a false discovery rate (FDR) of 5% for diabetes diagnosis and diabetes type separately.

References

1. Hansen TF, Banasik K, Erikstrup C, et al (2019) DBDS Genomic Cohort, a prospective and comprehensive resource for integrative and temporal analysis of genetic, environmental and lifestyle factors affecting health of blood donors. BMJ Open 9(6):e028401. https://doi.org/10.1136/BMJOPEN-2018-028401
2. Burgdorf KS, Simonsen J, Sundby A, et al (2017) Socio-demographic characteristics of Danish blood donors. PLOS ONE 12(2):e0169112. https://doi.org/10.1371/journal.pone.0169112
3. Carstensen B, Rønn PF, Jørgensen ME (2020) Prevalence, incidence and mortality of type 1 and type 2 diabetes in Denmark 1996–2016. BMJ Open Diabetes Res Care 8(1):e001071. https://doi.org/10.1136/BMJDRC-2019-001071
4. Schmidt M, Schmidt SAJ, Sandegaard JL, Ehrenstein V, Pedersen L, Sørensen HT (2015) The Danish National Patient Registry: a review of content, data quality, and research potential. Clin Epidemiol 7:449–490. https://doi.org/10.2147/CLEP.S91125
5. Pottegård A, Schmidt SAJ, Wallach-Kildemoes H, Sørensen HT, Hallas J, Schmidt M (2017) Data Resource Profile: The Danish National Prescription Registry. Int J Epidemiol 46(3):798–798f. https://doi.org/10.1093/ije/dyw213
6. Andersen JS, Olivarius NDF, Krasnik A (2011) The Danish National Health Service Register. Scand J Public Health 39(7 Suppl):34–37. https://doi.org/10.1177/1403494810394718
7. Erikstrup C, Sørensen E, Nielsen KR, et al (2022) Cohort Profile: The Danish Blood Donor Study. Int J Epidemiol dyac194. https://doi.org/10.1093/ije/dyac194
8. Privé F, Arbel J, Vilhjálmsson BJ (2020) LDpred2: better, faster, stronger. Bioinformatics 36(22–23):5424–5431. https://doi.org/10.1093/bioinformatics/btaa1029
9. Tramunt B, Smati S, Grandgeorge N, et al (2020) Sex differences in metabolic regulation and diabetes susceptibility. Diabetologia 63(3):453–461. https://doi.org/10.1007/s00125-019-05040-3
10. Reis JP, Loria CM, Sorlie PD, Park Y, Hollenbeck A, Schatzkin A (2011) Lifestyle Factors and Risk for New-Onset Diabetes. Ann Intern Med 155(5):292–299. https://doi.org/10.7326/0003-4819-155-5-201109060-00006
11. Navarro SL, Kantor ED, Song X, et al (2016) Factors Associated with Multiple Biomarkers of Systemic Inflammation. Cancer Epidemiol Biomarkers Prev 25(3):521–531. https://doi.org/10.1158/1055-9965.EPI-15-0956
12. Beyene HB, Olshansky G, Smith AAT, et al (2020) High-coverage plasma lipidomics reveals novel sex-specific lipidomic fingerprints of age and BMI: Evidence from two large population cohort studies. PLOS Biol 18(9):e3000870. https://doi.org/10.1371/journal.pbio.3000870
13. Enroth S, Hallmans G, Grankvist K, Gyllensten U (2016) Effects of Long-Term Storage Time and Original Sampling Month on Biobank Plasma Protein Concentrations. EBioMedicine 12:309–314. https://doi.org/10.1016/j.ebiom.2016.08.038
14. Kanabrocki EL, Sothern RB, Scheving LE, et al (1990) Reference values for circadian rhythms of 98 variables in clinically healthy men in the fifth decade of life. Chronobiol Int 7(5–6):445–461. https://doi.org/10.3109/07420529009059156
15. Kim K, Mall C, Taylor SL, et al (2014) Mealtime, Temporal, and Daily Variability of the Human Urinary and Plasma Metabolomes in a Tightly Controlled Environment. PLoS ONE 9(1):e86223. https://doi.org/10.1371/journal.pone.0086223
16. Brunzell JD, Rohlfing JJ (1986) The Effects of Diuretics and Adrenergic-Blocking Agents on Plasma Lipids. West J Med 145(2):210–218
17. van Raalte DH, Diamant M (2014) Steroid diabetes: from mechanism to treatment? 72(2)
18. McClain KM, Moore SC, Sampson JN, et al (2020) Preanalytical Sample Handling Conditions and Their Effects on the Human Serum Metabolome in Epidemiologic Studies. Am J Epidemiol 190(3):459–467. https://doi.org/10.1093/aje/kwaa202
19. Scott R, Langenberg C, Sharp S, et al (2013) The link between Family History and risk of Type 2 Diabetes is Not Explained by Anthropometric, Lifestyle or Genetic Risk Factors: the EPIC-InterAct Study. Diabetologia 56(1):60–69. https://doi.org/10.1007/s00125-012-2715-x
20. Pedersen CB (2011) The Danish Civil Registration System. Scand J Public Health 39(7_suppl):22–25. https://doi.org/10.1177/1403494810387965
21. van Buuren S (2016) Multiple imputation of discrete and continuous data by fully conditional

specification. 16(3):219–242. https://doi.org/10.1177/0962280206074463
22. Marshall A, Altman DG, Holder RL, Royston P (2009) Combining estimates of interest in prognostic modelling studies after multiple imputation: Current practice and guidelines. BMC Med Res Methodol 9(1):1–8. https://doi.org/10.1186/1471-2288-9-57/TABLES/2
23. Tabák AG, Jokela M, Akbaraly TN, Brunner EJ, Kivimäki M, Witte DR (2009) Trajectories of glycaemia, insulin sensitivity, and insulin secretion before diagnosis of type 2 diabetes: an analysis from the Whitehall II study. The Lancet 373(9682):2215–2221. https://doi.org/10.1016/S0140-6736(09)60619-X
24. Porta M, Curletto G, Cipullo D, et al (2014) Estimating the Delay Between Onset and Diagnosis of Type 2 Diabetes From the Time Course of Retinopathy Prevalence. Diabetes Care 37(6):1668–1674. https://doi.org/10.2337/dc13-2101
25. Harrell FE (2001) Regression Modeling Strategies: With Applications to Linear Models, Logistic Regression, and Survival Analysis. Springer, New York, NY
26. Hobohm U, Scharf M, Schneider R, Sander C (1992) Selection of representative protein data sets. Protein Sci Publ Protein Soc 1(3):409–417

ESM Figures

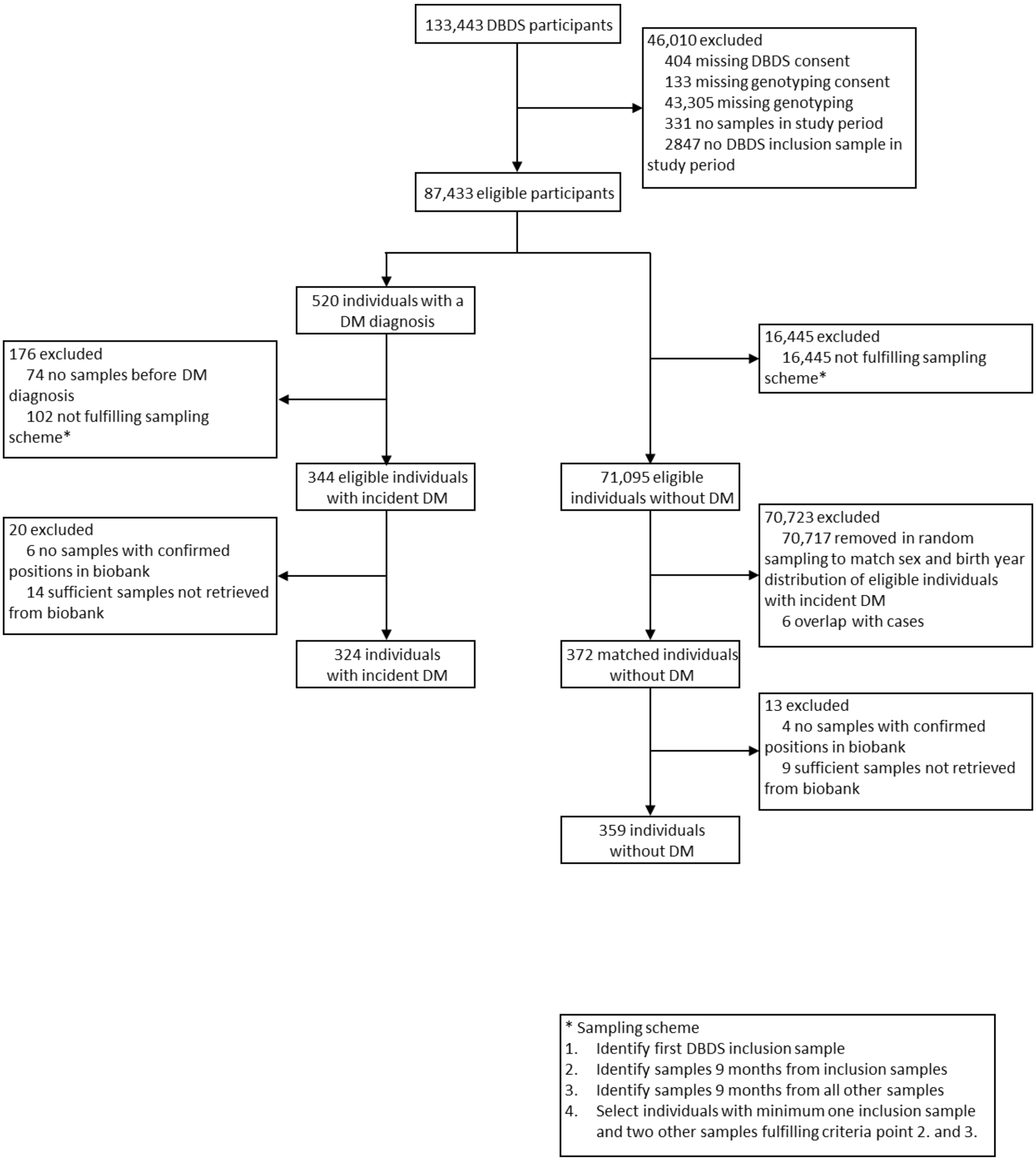

ESM Fig. 1 Flow chart for the selection of incident diabetes cases and individuals without diabetes.

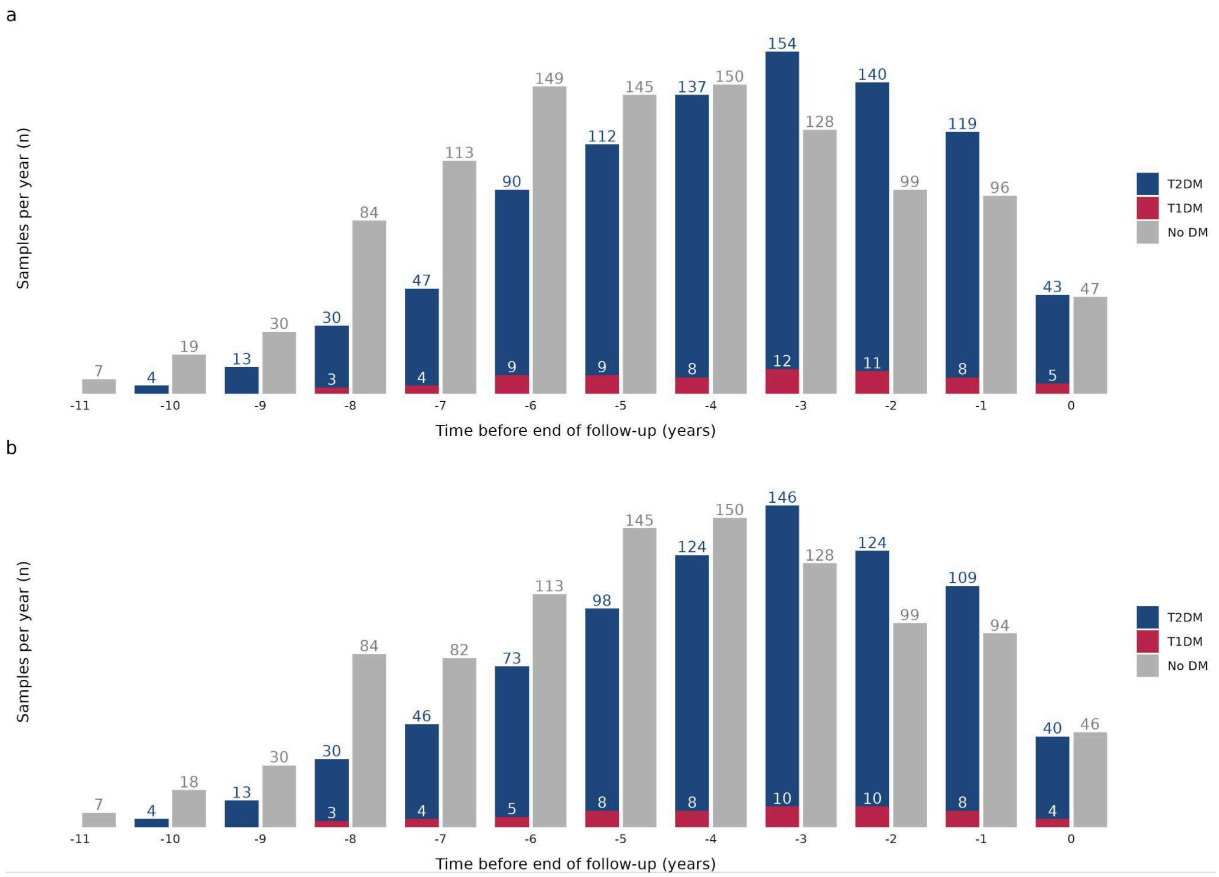

ESM Fig. 2 Number of samples for each year before the end of follow-up for proteins (a) and metabolites and lipoprotein particles (b). The end of follow-up is defined as the time from diabetes diagnosis (individuals with an incident diagnosis of type 1 diabetes or type 2 diabetes) or the end of the study period on 31 December 2016 (individuals without diabetes).

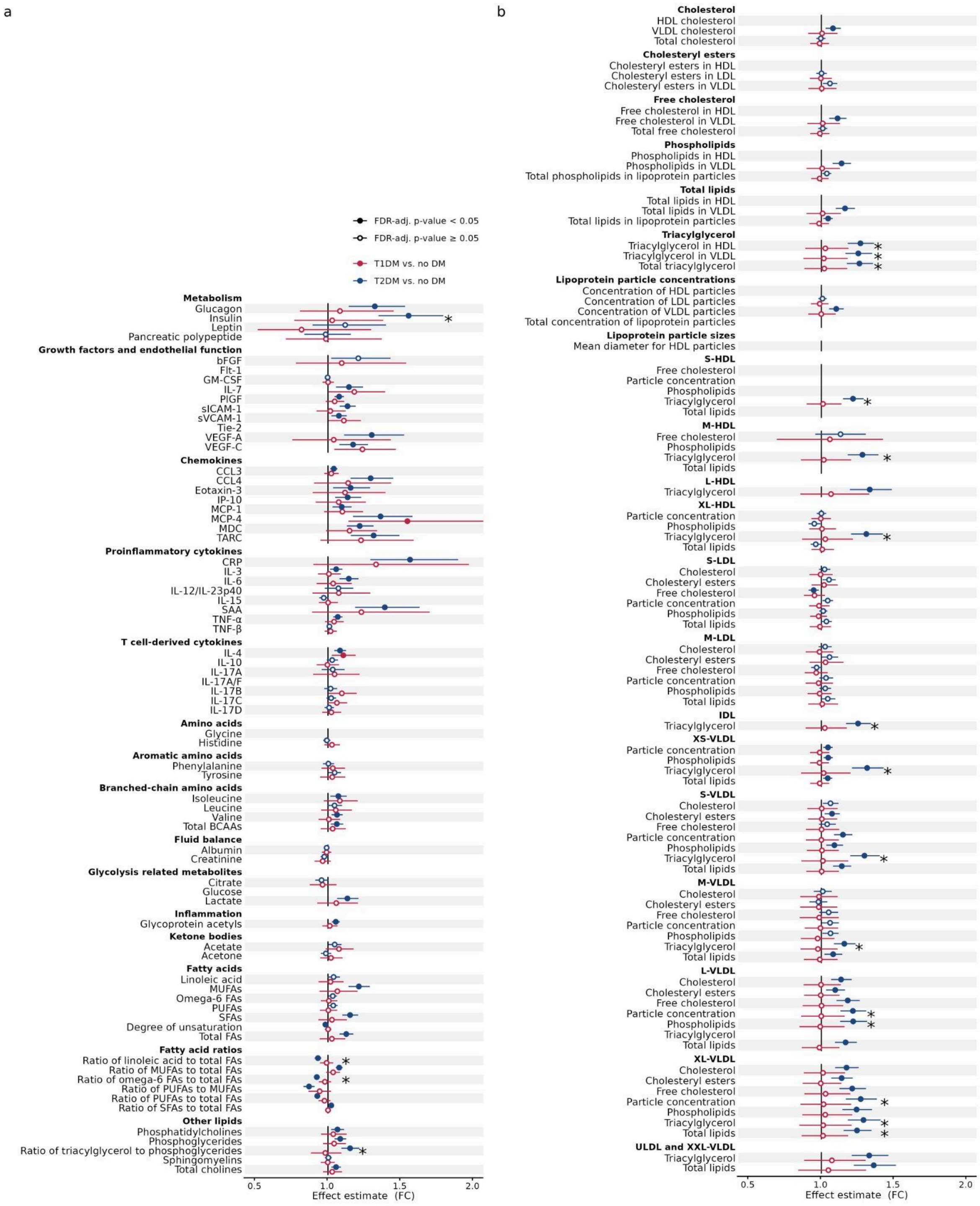

ESM Fig. 3 Biomarker-specific effect estimates for type 1 and type 2 diabetes separately obtained using mixed-effects models (fold change, FC) are shown for proteins (**a**), metabolites (**a, b**) and lipoprotein particles (**b**). All estimates are shown as point estimates and 95% CI. Comparisons of the type 2 diabetes group to the non-diabetes group are shown in blue and the type 1 diabetes group to the non-diabetes group in red. Estimates for biomarkers with a significant test of interaction (ANOVA FDR-adj. p value < 0.05) are not shown but can be found in ESM Figure 4. Significant differences between the type 1 diabetes and type 2 diabetes groups are shown with an asterisk. Significant estimates (FDR-adjusted p value < 0.05) are indicated by filled circles; non-significant estimates are indicated by open circles.

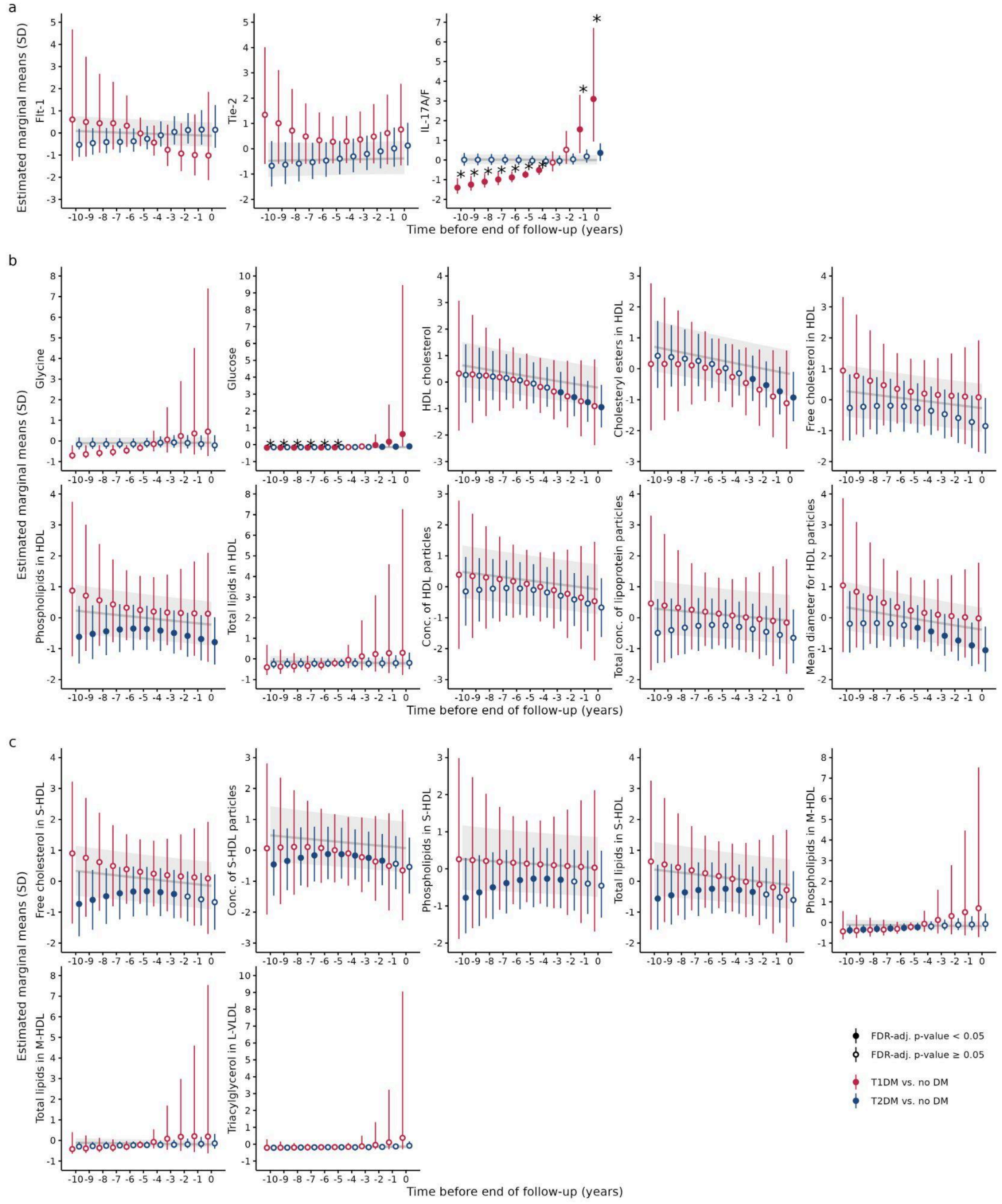

ESM Fig. 4 Per-year estimated marginal means for diabetes type-specific temporally changing proteins (**a**), metabolites (**b**), and lipoprotein particles (**c**). Estimated marginal means and 95% confidence interval for the type 1 diabetes (red) and type 2 diabetes (blue) groups are shown for each year before the end of follow-up at time zero (diabetes diagnosis for incident diabetes cases and the end of study period for individuals without diabetes), while the non-diabetes group is shown as a line with shaded area. Values are z score normalised to ease visualisation; hence one unit difference corresponds to one standard deviation (SD). See the exact EMMs in ESM Table 4. Point fill indicates significance (FDR-adjusted p value < 0.05) (filled) or non-significant (open). Significant differences (FDR-adjusted p value < 0.05) between the two diabetes groups are shown with an asterisk.

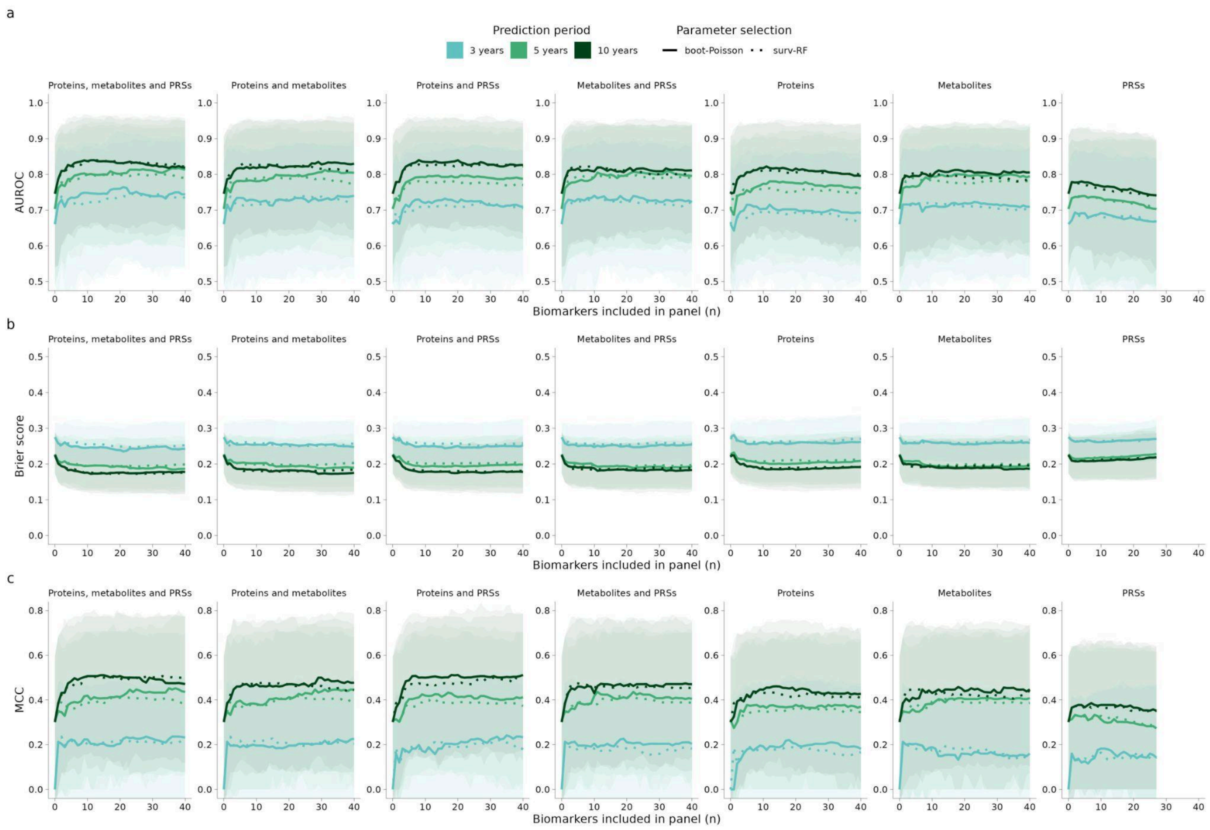

ESM Fig. 5 Poisson model performance for 41 panels as assessed by AUROC (**a**), Brier score (**b**) and MCC (**c**). Median and 2.5-97.5% percentile intervals for the 41 panels assessed for each combination of molecular types (proteins, metabolites, and PRSs) together with confounding and technical covariates. Parameter selection methods are indicated by line type and prediction period by colour. MCC is calculated for cumulative risk cut-off=0.5.

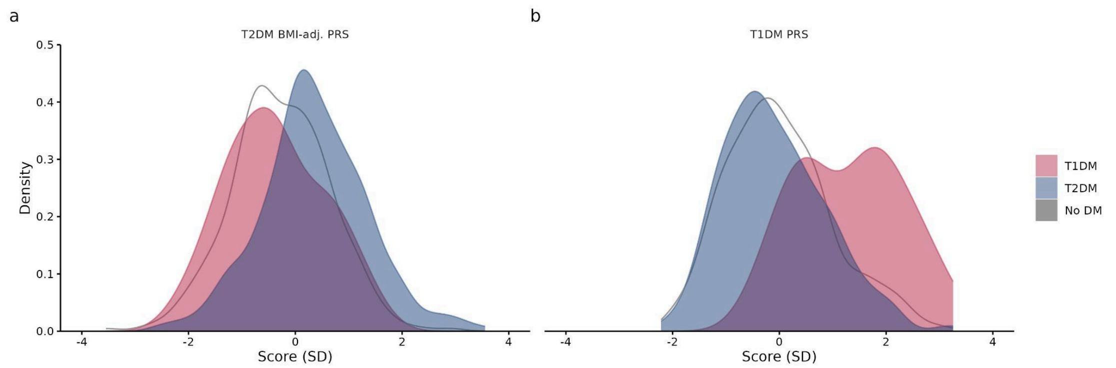

ESM Fig. 6 Distributions of type 1 diabetes (**a**) and BMI-adj. type 2 diabetes (**b**) PRSs for individuals without diabetes (black outline), individuals with incident type 1 diabetes (red fill), and individuals with incident type 2 diabetes (blue fill), as identified in the DBDS diabetes registry. Values have been *z* score normalised, hence one unit difference corresponds to one SD.

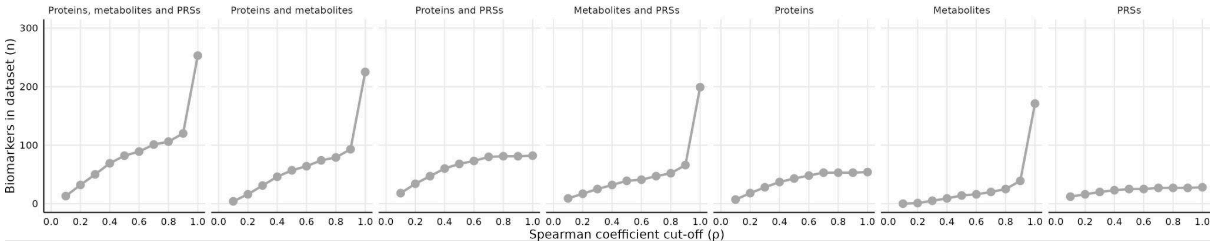

ESM Fig. 7 Number of markers included after removal of collinearity using the Hobohm II algorithm. The number of markers included in the seven combinations of molecular data (protein, metabolites, and PRSs). Correlation is calculated using Spearman correlation coefficient. The number of markers was evaluated at ten thresholds of correlation coefficients.

DBDS Genomic Consortium members

Member	Affiliation(s)
Karina Banasik	Novo Nordisk Foundation Center for Protein Research, Faculty of Health and Medical Sciences, University of Copenhagen, Copenhagen, Denmark
Jakob Bay	Department of Clinical Immunology, Zealand University Hospital, Køge, Denmark
Jens Kjærgaard Boldsen	Department of Clinical Immunology, Aarhus University Hospital, Aarhus, Denmark
Thorsten Brodersen	Department of Clinical Immunology, Zealand University Hospital, Køge, Denmark
Søren Brunak	Novo Nordisk Foundation Center for Protein Research, Faculty of Health and Medical Sciences, University of Copenhagen, Copenhagen, Denmark
Kristoffer Burgdorf	Novo Nordisk Foundation Center for Protein Research, Faculty of Health and Medical Sciences, University of Copenhagen, Copenhagen, Denmark
Mona Ameri Chalmer	Danish Headache Center, Department of Neurology, Copenhagen University Hospital, Rigshospitalet-Glostrup, Copenhagen, Denmark
Maria Didriksen	Department of Clinical Immunology, Copenhagen University Hospital, Rigshospitalet, Copenhagen, Denmark
Khoa Manh Dinh	Department of Clinical Immunology, Aarhus University Hospital, Aarhus, Denmark
Joseph Dowsett	Department of Clinical Immunology, Copenhagen University Hospital, Rigshospitalet, Copenhagen, Denmark
Christian Erikstrup	Department of Clinical Immunology, Aarhus University Hospital, Aarhus, Denmark<br>Department of Clinical Medicine, Health, Aarhus University, Aarhus, Denmark
Bjarke Feenstra	Department of Clinical Immunology, Copenhagen University Hospital, Rigshospitalet, Copenhagen, Denmark<br>Department of Epidemiology Research, Statens Serum Institut, Copenhagen, Denmark
Frank Geller	Department of Clinical Immunology, Copenhagen University Hospital, Rigshospitalet, Copenhagen, Denmark<br>Department of Epidemiology Research, Statens Serum Institut, Copenhagen, Denmark
Daniel Gudbjartsson	deCODE Genetics, Reykjavik, Iceland
Thomas Folkmann Hansen	Danish Headache Center, Department of Neurology, Copenhagen University Hospital, Rigshospitalet-Glostrup, Copenhagen, Denmark
Lotte Hindhede	Department of Clinical Immunology, Aarhus University Hospital, Aarhus, Denmark
Henrik Hjalgrim	Danish Cancer Society Research Center, Copenhagen, Denmark<br>Department of Epidemiology Research, Statens Serum Institut, Copenhagen, Denmark
Rikke Louise Jacobsen	Department of Clinical Immunology, Copenhagen University Hospital, Rigshospitalet, Copenhagen, Denmark
Gregor Jemec	Department of Dermatology, Zealand University Hospital, Roskilde, Denmark

Bitten Aagaard Jensen	Department of Clinical Immunology, Aalborg University Hospital, Aalborg, Denmark
Katrine Kaspersen	Department of Clinical Immunology, Aarhus University Hospital, Aarhus, Denmark
Bertram Dalskov Kjerulff	Department of Clinical Immunology, Aarhus University Hospital, Aarhus, Denmark
Lisette Kogelman	Danish Headache Center, Department of Neurology, Copenhagen University Hospital, Rigshospitalet-Glostrup, Copenhagen, Denmark
Margit HørupLarsen	Department of Clinical Immunology, Copenhagen University Hospital, Rigshospitalet, Copenhagen, Denmark
Ioannis Louloudis	Novo Nordisk Foundation Center for Protein Research, Faculty of Health and Medical Sciences, University of Copenhagen, Copenhagen, Denmark
Agnete Troen Lundgaard	Novo Nordisk Foundation Center for Protein Research, Faculty of Health and Medical Sciences, University of Copenhagen, Copenhagen, Denmark
Susan Mikkelsen	Department of Clinical Immunology, Aarhus University Hospital, Aarhus, Denmark
Christina Mikkelsen	Department of Clinical Immunology, Copenhagen University Hospital, Rigshospitalet, Copenhagen, Denmark
Ioanna Nissen	Department of Clinical Immunology, Copenhagen University Hospital, Rigshospitalet, Copenhagen, Denmark
Mette Nyegaard	Department of Health Science and Technology, Faculty of Medicine, Aalborg University, Aalborg, Denmark
Sisse Rye Ostrowski	Department of Clinical Immunology, Copenhagen University Hospital, Rigshospitalet, Copenhagen, Denmark<br>Department of Clinical Medicine, Faculty of Health and Medical Sciences, University of Copenhagen, Copenhagen, Denmark
Ole Birger Pedersen	Department of Clinical Immunology, Zealand University Hospital, Køge, Denmark<br>Department of Clinical Medicine, Faculty of Health and Medical Sciences, University of Copenhagen, Copenhagen, Denmark
Alexander Pil Henriksen	Novo Nordisk Foundation Center for Protein Research, Faculty of Health and Medical Sciences, University of Copenhagen, Copenhagen, Denmark
Palle Duun Rohde	Department of Health Science and Technology, Faculty of Medicine, Aalborg University, Aalborg, Denmark
Klaus Rostgaard	Danish Cancer Society Research Center, Copenhagen, Denmark<br>Department of Epidemiology Research, Statens Serum Institut, Copenhagen, Denmark
Michael Schwinn	Department of Clinical Immunology, Copenhagen University Hospital, Rigshospitalet, Copenhagen, Denmark
Kari Stefansson	deCODE Genetics, Reykjavik, Iceland
Hreinn Stefánsson	deCODE Genetics, Reykjavik, Iceland
Erik Sørensen	Department of Clinical Immunology, Copenhagen University Hospital, Rigshospitalet, Copenhagen, Denmark
Unnur Þorsteinsdóttir	deCODE Genetics, Reykjavik, Iceland
Lise Wegner Thørner	Department of Clinical Immunology, Copenhagen University Hospital, Rigshospitalet, Copenhagen, Denmark

Mie Topholm Bruun	Department of Clinical Immunology, Odense University Hospital, Odense, Denmark
Henrik Ullum	Statens Serum Institut, Copenhagen, Denmark
Thomas Werge	Institute of Biological Psychiatry, Mental Health Centre, Sct. Hans, Copenhagen University Hospital, Roskilde, Denmark<br>Department of Clinical Medicine, Faculty of Health and Medical Sciences, University of Copenhagen, Copenhagen, Denmark
David Westergaard	Novo Nordisk Foundation Center for Protein Research, Faculty of Health and Medical Sciences, University of Copenhagen, Copenhagen, Denmark
